# Uncovering New Diversity of Photosynthetic Microorganisms from the Mediterranean Region

**DOI:** 10.3390/microorganisms10081571

**Published:** 2022-08-04

**Authors:** Urania Lortou, Emmanuel Panteris, Spyros Gkelis

**Affiliations:** Department of Botany, School of Biology, Faculty of Sciences, Aristotle University of Thessaloniki, GR-541 24 Thessaloniki, Greece

**Keywords:** chlorophyta, microalgae, molecular systematics, phylogeny, taxonomy, ultrastructure

## Abstract

In the large and morphologically diverse phylum of Chlorophyta, new taxa are discovered every year and their phylogenetic relationships are reconstructed by the incorporation of molecular phylogenetic methods into traditional taxonomy. Herein, we aim to contribute to the photosynthetic microorganisms’ diversity knowledge in the Mediterranean area, a relatively unexplored ecoregion with high diversity. Based on a polyphasic approach, 18 Chlorophyta isolates were investigated and characterized. Morphological characteristics and ultrastructure, the phylogeny based on 18S rRNA gene (small subunit ribosomal RNA), 18S–28S internal transcribed spacer (ITS region), and the ribulose-1,5-bisphosphate carboxylase/oxygenase large subunit region (*rbcL* gene), support establishing four new genera (*Nomia*, *Ava*, *Akraea*, *Lilaea*) and five new species (*Spongiosarcinopsis limneus*, *N. picochloropsia*, *Av. limnothalassea*, *Ak. chliaropsychia*, and *L. pamvotia*) belonging to orders Sphaeropleales, Chlorellales, and Chlamydomonadales. For some of them, this is the first report of their occurrence in specific aquatic environments.

## 1. Introduction

Phylum Chlorophyta, one of the significant groups of photosynthetic microorganisms is also one of the most abundant in microphytoplankton. It is an early-diverging lineage of the Viridiplantae and is morphologically, cytologically, and ecologically diverse [1]. Chlorophyta comprise a wide diversity of freshwater, marine, and even terrestrial green algae [2]. Their role in the ecosystem’s balance worldwide is outstanding [1,3]. The core includes the clades Chlorodendrophyceae, Ulvophyceae, Trebouxiophyceae, and Chlorophyceae, the latter being dominant among Chlorophyta [4]. To date, Chlorophyta contain approximately 6730 species of which 3630 belong to the monophyletic class Chlorophyceae [5]. Nevertheless, the diversity of photosynthetic microeukaryotes is still underestimated and our understanding of their taxonomic and phylogenetic relationships is deficient [1,6,7]. Moreover, most of the studies on Chlorophyta globally, investigate macroalgae diversity [8,9,10,11].

New taxa and lineages of green algae are discovered every year, due to the use of molecular tools in combination with the traditional morphological taxonomy. For many years the identification of microscopic green algae was mainly based on the recognition of morphological characteristics such as the shape of the cells, position of chloroplasts, presence or absence of pyrenoids, and type of reproduction [1]. The lack of obvious traits, the high degree of variability of some of the observable features of species, and the phenotypic plasticity that several microorganisms exhibit led to a large number of misidentifications and difficulties in the delimitation of species [2,12,13]. Thus, the combination of approaches thrived among phycologists since more robust results could be obtained [7,14] and that led to a total revision of taxonomic lineages such as classes, orders, and families [15].

According to Singh and Saxena [16], territories around the Mediterranean Sea are more suitable for algae cultivation than those used in industry owing to the favorable climate. Nevertheless, the Mediterranean region is considered as a relatively unexplored area regarding the diversity of microalgae. Culture-dependent approaches to studying the molecular diversity and phylogeny of microalgae derived from Mediterranean aquatic environments are limited [17,18,19,20]. The plethora of studies is limited to morphological descriptions of microalgae and/or surveys on the ecological status of ecosystems [21,22,23]. Furthermore, in several studies, molecular cloning techniques are used to identify microalgae in freshwater ecosystems [24,25,26]. Some studies have as central content the biotechnological potential of green algae [27,28,29,30]. Greece is a country in the Mediterranean with an immoderate level of diversity and endemism of species [31], a fact which has led to the frequent recording of new taxa of photosynthetic microorganisms. This study aims to characterize in detail the diversity of green algae, previously isolated from different habitats in Greece [18], by combining molecular phylogeny, morphology, and ultrastructural data.

## 2. Materials and Methods

### 2.1. Sample Collection, Strains Isolation, and Growth

Eighteen strains were isolated from surface water samples between the years 2010 and 2017. The collection spots covered freshwaters (Lakes Doirani, Koronia, Pamvotis, and Volvi), a thermal spring (Agkistro), a lagoon (Kalochori), and a water pond at the Aristotle University of Thessaloniki (AUTH), in Greece (Table 1, Figure S1). For a description of the lakes and the thermal spring see [18].

The Kalochori Lagoon is part of one of the most important ecosystems in Greece, the Axios Delta National Park. Kalochori village occupies the lowlands between the west side of the city of Thessaloniki and the delta of the Gallikos River. The wetland complex includes the Kalochori Lagoon, the estuary of the Gallikos River, the delta of the Axios River, the estuary of the Loudias River, the delta of the Aliakmon River, and the Alyki Kitrous wetlands. The lagoon which constitutes the northern gate of the National Park, being at the edge of a large urban center and near the industrial area and the harbor, is a coastal wetland of special interest hosting ecologically important populations of birds and other organisms throughout the year [32]. Water temperature range between 10.4 and 26.8 °C, salinity and pH near the bottom varies between 34.1 and 37.0 psu and 7.64 and 9.4, respectively, during spring-summer (March, June) and autumn-winter (September, December) [33].

Water samples were collected from the surface layer (0–0.5 m) of inshore sites, details are given in [34], and in one case (Agkistro), algal mat samples attached to stones were collected after carefully scratching with a sterile scalpel on the mat and placing the detached mat into 50 mL sterile polyethylene vessels. Strain isolation was performed as described by Gkelis et. al. [35]. Strains were cultured in liquid BG-11 medium with nitrogen [36] and maintained in the same medium by regular sub-culturing every 2 to 4 weeks. Cultures are maintained in the Aristotle University of Thessaloniki MicroAlgae and Cyanobacteria Culture Collection (TAU-MAC) [37] at 20 ± 2 °C, at a photosynthetic photon flux density of 20 μmol m^−2^s^−1^ using cool white light fluorescent tubes (Sylvania Standard F36W/154-T8, SLI) in a 16:8 h light: dark cycle.

Preliminary characterization of five strains (TAU-MAC 0910, 1010, 3310, 3510, and 0215) investigated in the present work, had previously been performed [18].

### 2.2. Morphology and Ultrastructure

For light microscopy, Chlorophyta strains were examined under a Zeiss AxioImager.Z2 (Carl Zeiss, Oberkochen, Germany) light microscope equipped with an AxioCam MRc5 digital camera (Carl Zeiss, Germany) and DIC optics. Mean cell dimensions were calculated after measuring the dimension of at least 50 cells per strain, using AxioVision Rel. 4.8.2 software (Carl Zeiss). Strains were identified and detailed morphological descriptions of the novel genera and species were performed based on special taxonomic articles and books [38,39,40,41,42,43,44,45,46]. The International Code of Nomenclature for algae, fungi, and plants (ICN) was used for the names of new taxa [47].

For Transmission Electron Microscopy (TEM), a mixed sample of each strain, from cultures of 10 days, 1 month, and 4 months after inoculation, was prepared. Cells were collected by gentle centrifugation and embedded in 2% *w*/*v* low melting point agarose (ABT, Madrid, Spain), by mixing 10 μL of algal pellet with 10 μL molten agarose. The algal cells, encapsulated in solidified agarose blocks, were fixed in 3% (*v*/*v*) glutaraldehyde (PolySciences, Niles, IL, USA) in 50 mM sodium cacodylate buffer (pH 7) for 4 h at room temperature, then washed 3 × 15 min with the same buffer and post-fixed in 1% (*w*/*v*) osmium tetroxide overnight at 4 °C. After washing as previously, the samples were dehydrated at room temperature in a graded acetone series followed by 2 × 30 min of propylenoxide treatment at 4 °C. Dehydrated cells were embedded in 3:1, 2:1 and 1:1 propylenoxide-Spurr’s resin (PolySciences, Niles, IL, USA) for 24 h at 4 °C each step. Afterward, the 1:1 propylenoxide-resin mixture was left overnight to evaporate at room temperature, then fresh pure resin was added, and the samples were put for 4 h on a rotator. Finally, the samples with resin were put at 65 °C for 48 h for polymerization. Ultrathin sections (60–80 nm) were cut with a Reichert–Jung ULTRACUT E ultramicrotome (Reichert–Jung Optical Company, Vienna, Austria), double stained with 4% (*w*/*v*) uranyl acetate and 1% (*w*/*v*) lead citrate, and examined with a JEOL JEM 1011 (JEOL Ltd., Tokyo, Japan) TEM at 80 kV and electron micrographs were taken with a Gatan ES500W digital camera (Pleasanton, CA, USA).

### 2.3. DNA Extraction, Amplification, and Sequencing

The Cetyl Trimethylammonium Bromide (CTAB) based protocol [48] was used to extract total genomic DNA from algae monocultures in order to amplify regions with phylogenetic importance as described previously [18]. In brief, isolated genomic DNA was used as a template to amplify the 18S rRNA gene, ITS region, and sequence of *rbcL* gene following the PCR conditions given in Table S1. Several additional sets of primers (Table S1) were used in order to attain amplification of the three genetic loci mentioned above for all strains. Thermal cycling was carried out using an Eppendorf MasterCycler Pro (Eppendorf). The amplified products were confirmed using electrophoresis on 1.5% (*w*/*v*) agarose gel in 1× TAE buffer, visualized with Midory Green (Nippon Genetics Europe GmbH, Düren, Germany) under UV light, and purified using the Monarch^®^ PCR and DNA Cleanup Kit (New England BioLabs, Ipswich, MA, USA) kit. The purified products were sequenced with a combination of primers of Table S1 using the ABI 3730xl DNA Analyzer. Forward and reverse sequences were visually inspected, assembled, and edited manually using BioEdit (Ibis Biosciences 1997–2015©). ITS region and *rbcL* gene sequence data were checked for chimeras using the RDPII chimera detection [49]. The nucleotide sequences of the partial 18S rRNA, total ITS region, and partial *rbcL* gene from strains in this study have been deposited in the GenBank database [50] with accession numbers OK641927-OK641941, OK642359-OK642371, and OK626420-OK626438, respectively. The ITS region sequence of TAU-MAC 3617 and *rbcL* gene sequence of TAU-MAC 0215 could not be obtained.

### 2.4. Phylogenetic Analysis

For the detection of closest relatives, sequences obtained in this study and in a previous study [18] were compared by a similarity search with the BLASTn algorithm (National Center for Biotechnology Information-GenBank). The dataset for 18S rRNA phylogenetic analysis was generated by including some of the closest relatives along with sequences from representative families of the orders Sphaeropleales, Chlorellales, Chlamydomonadales, and Chaetophorales (classes Chlorophyceae and Trebouxiophyceae). In order to evaluate relationships inside orders, ITS region and *rbcL* gene phylogenetic trees were constructed for each order separately using sequences belonging to major families and their related sister genera to our strains. TAU-MAC strains belonging to orders Chlamydomonadales and Chaetophorales were included in the same tree. Sequences were aligned using the ClustalW [51] or MUSCLE [52] alignment utility through MEGA7 v.7.0 software [53] depending on the dataset. The sequence data of the 18S-28S ITS region and *rbcL* gene were used individually for independent phylogenetic inference. Phylogenetic analyses were performed using Maximum Parsimony (MP), Maximum Likelihood (ML), and Bayesian inference (BI). The ML, using Gamma distributed with Invariant sites (G+I) upon default parameters and MP analyses, was implemented in MEGA7 and the confidence of the tree topologies was checked using bootstrap analyses (1000 replicates). Using the jModelTest v2.1.4 [54], the GTR+I+G model was determined as the most appropriate based on both the Bayesian and Akaike information criteria and was used for all ML and BI analyses (18S rRNA, ITS region, *rbcL* gene). Bayesian phylogenetic analyses were conducted using MrBayes 3.2.7 [55] with 10,000,000 generations of Markov chain Monte Carlo iterations (MCMC), discarding the first 25% as burn-in and the following datasets were sampled every 1000th generation.

## 3. Results and Discussion

**Figure 1:**
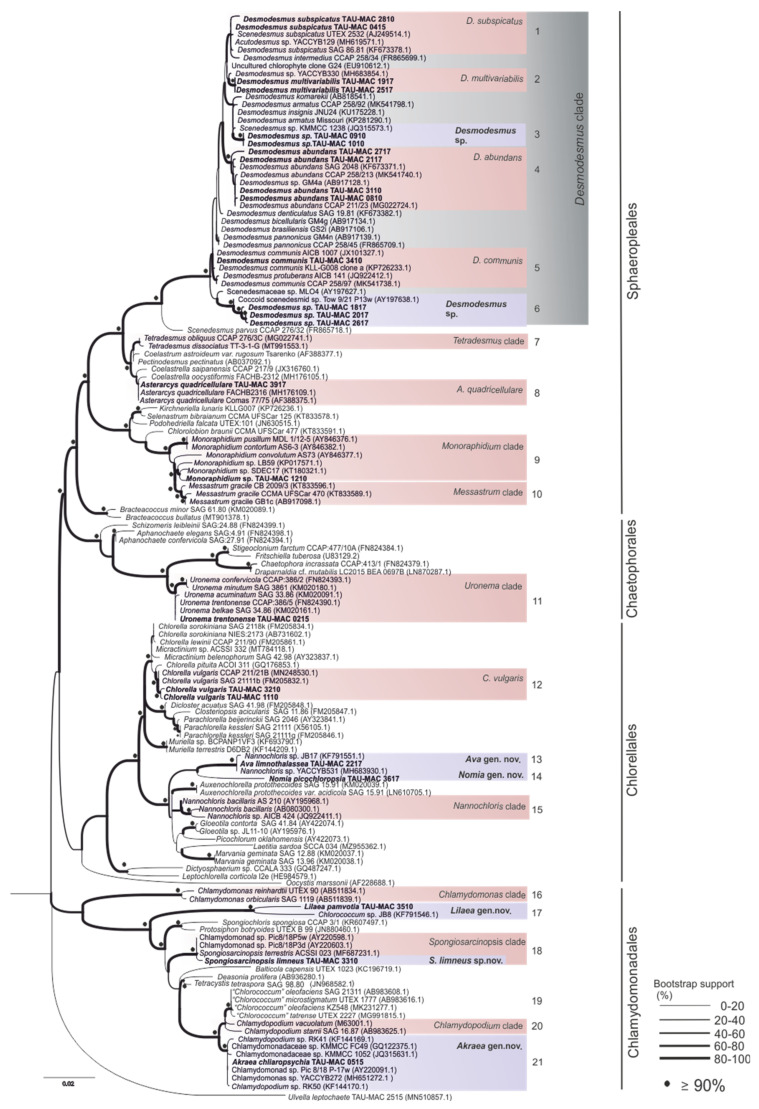
Phylogenetic tree of relationships of the TAU-MAC green algae strains based on 18S rRNA (c. 1700 bp) gene including Chlorophyceae and Trebouxiophyceae. Line thickness at branches represents bootstrap support values (bootstrap support for ML and MP analysis). The numbers in parentheses are GenBank accession numbers.

**Figure 2:**
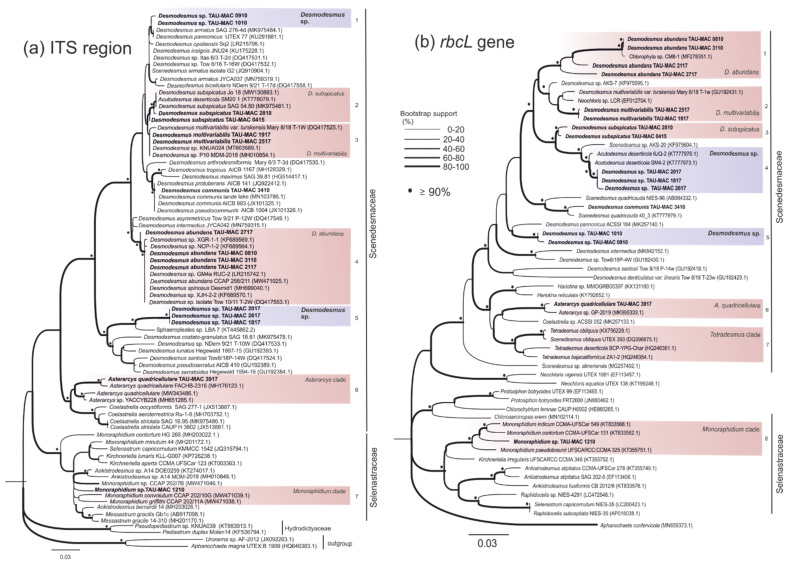
Phylogenetic relationships of the Sphaeropleales TAU-MAC strains based on ITS region (c. 500 bp) and *rbcL* gene (c. 1050 bp) genes including Scenedesmaceae and Selenastraceae families. Line thickness at branches (see explanation in figure) represents bootstrap support values (bootstrap support for ML and MP analysis). The numbers in parentheses are GenBank accession numbers.

**Figure 3:**
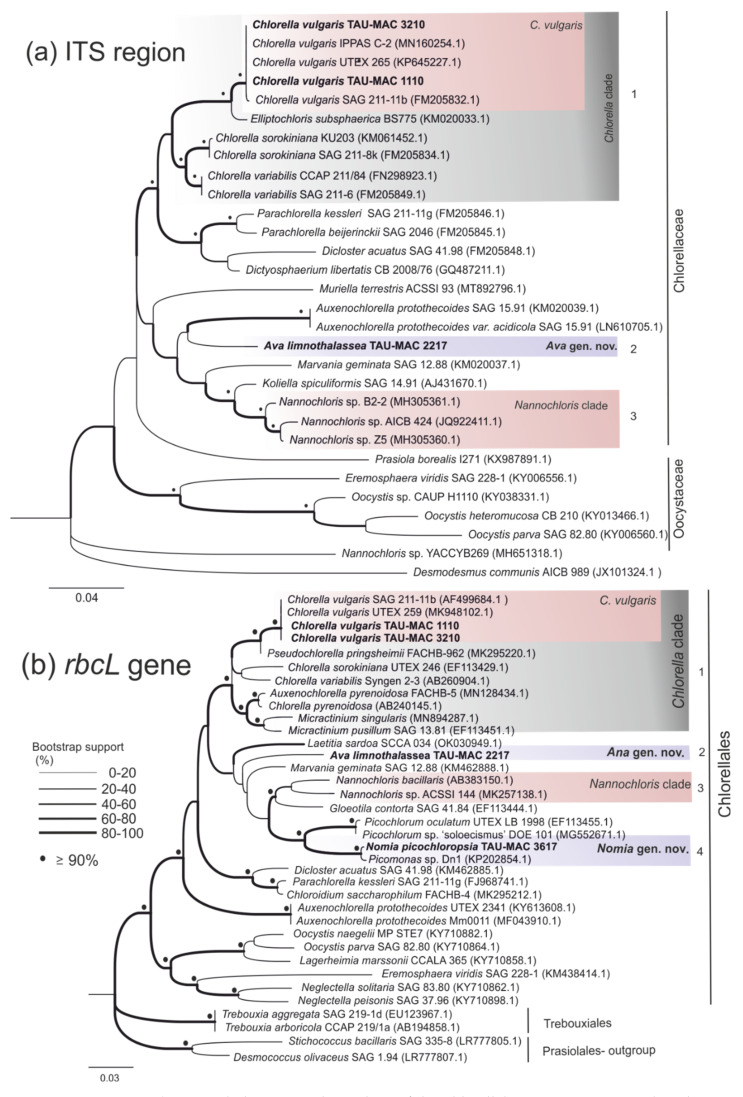
Phylogenetic relationships of the Chlorellales TAU-MAC strains based on ITS region (c. 500bp) and *rbcL* gene (c. 1050bp) genes. Line thickness at branches (see explanation in figure) represents bootstrap support values (bootstrap support for ML and MP analysis). The numbers in parentheses are GenBank accession numbers.

**Figure 4:**
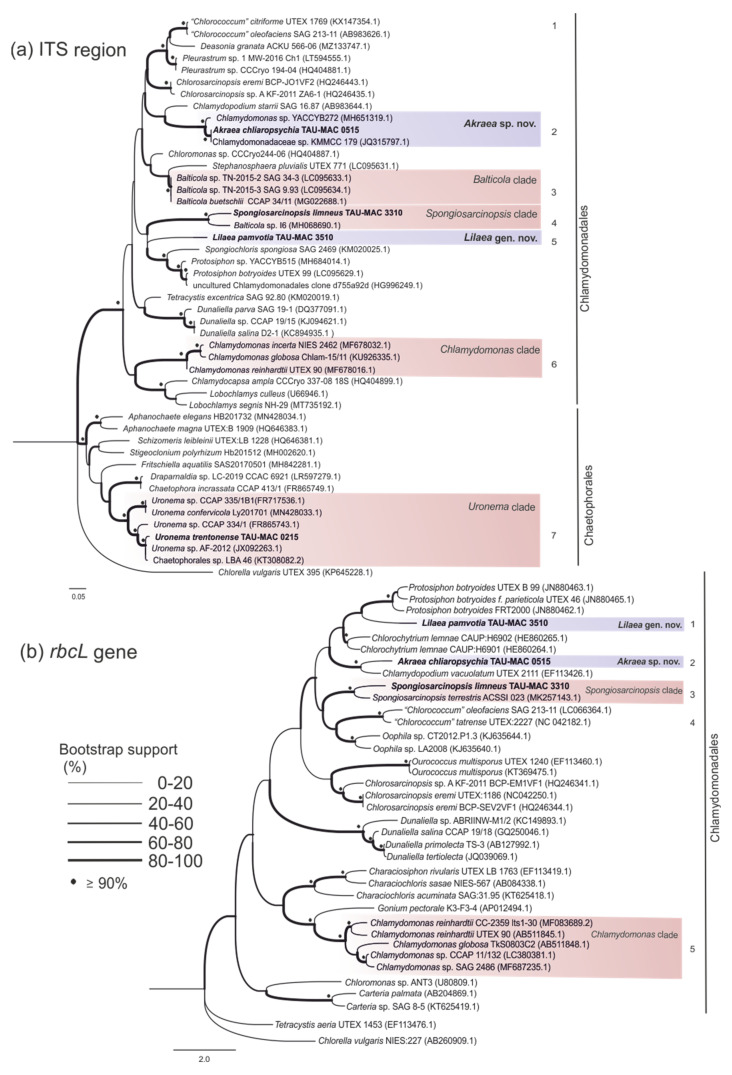
Phylogenetic relationships of the Chlamydomonadales TAU-MAC strains based on (**a**) ITS region (c. 500bp) and (**b**) rbcL gene (c. 1050bp) genes. Line thickness at branches (see explanation in figure) represents bootstrap support values (bootstrap support for ML and MP analysis). The numbers in parentheses are GenBank accession numbers.

**Figure 5:**
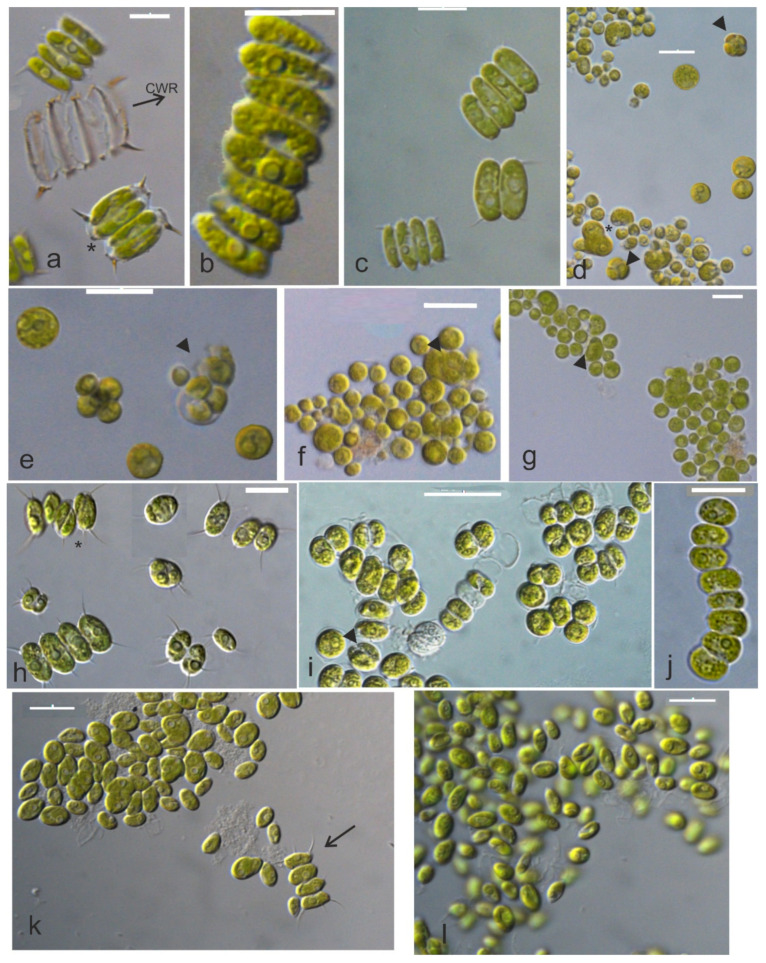
Light micrographs of strains *Desmodesmus* sp. TAU-MAC 0910 (**a**,**b**) and 1010 (**c**), *Desmodesmus* sp. 1817 (**d**), 2017 (**e**,**f**) and 2617 (**g**), *Desmodesmus subspicatus* 0415 (**h**), *Desmodesmus multivariabilis* 1917 (**i**) and 2517 (**j**), *Desmodesmus abundans* 2117 (**k**) and 2717 (**l**); (**a**): a mature four-celled coenobium, cell wall ornaments (observable from cell wall residues, CWR) and a cell division of mother cell into two daughter cells (asterisk). (**b**): an eight-celled coenobium. (**c**): mature two- and four-celled coenobia. (**d**): young and mature cells, autosporangia (arrowheads), and a cell division of the mother cell into two daughter cells (asterisk). (**e**,**f**): young and mature cells and aplanosporia (arrowhead). (**g**): young and mature solitary cells and aplanosporia (arrowhead). (**h**): coenobia with numerous spines and a cell division of mother cell into two daughter cells (asterisk). (**i**): solitary cells and two-celled coenobia and aplanosporia (arrowhead). (**j**): an eight-celled coenobium. (**k**): mostly solitary cells and four-celled coenobium with spines (arrow). (**l**): solitary cells. All images except (**g**), are with DIC optics. Bars, 10 μm.

**Figure 6:**
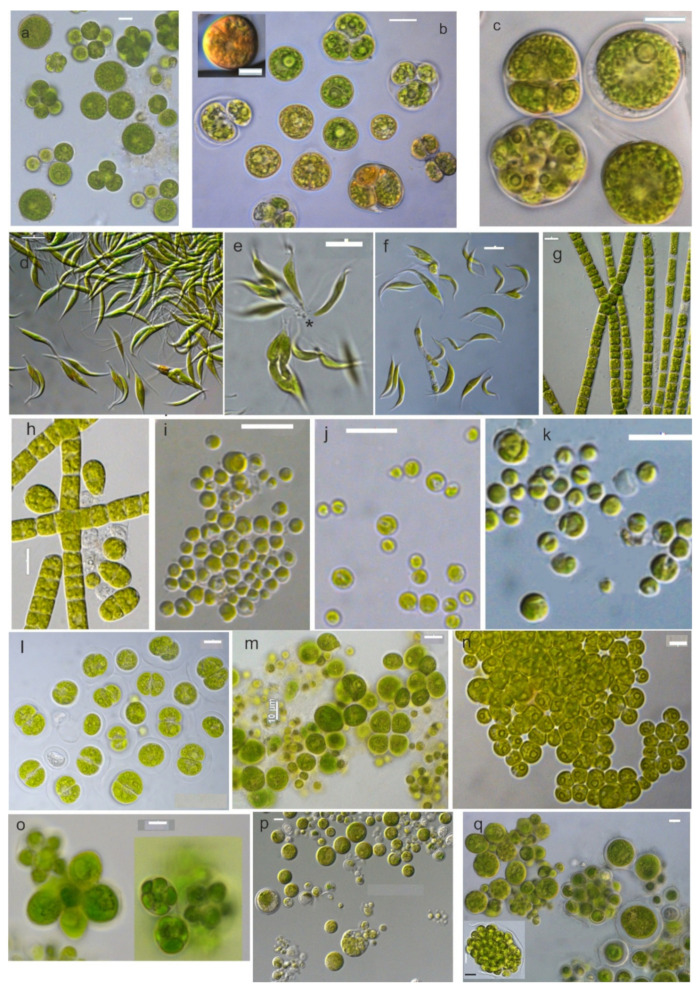
Light micrographs of strains *Asterarcys quadricellulare* TAU-MAC 3917 (**a**–**c**), *Monoraphidium* sp. TAU-MAC 1210 (**d**–**f**), *Uronema trentonense* TAU-MAC 0215 (**g**,**h**), *Ava limnothalassea* TAU-MAC 2217 (**i**,**j**), *Nomia picochloropsia* TAU-MAC 3617 (**k**), *Spongiosarcinopsis limneus* TAU-MAC 3310 (**l**,**m**), *Lilaea pamvotia* TAU-MAC 3510 (**n**,**o**), *Akraea chliaropsychia* TAU-MAC 0515 (**p**,**q**); (**a**–**c**): solitary cells, colonies of randomly arranged cells (arrow), autosporangia (arrowheads) and accumulation of pigments that were detected by color change (asterisk) (insert in (**b**)). (**d**–**f**): several different cell shapes, lunate to sigmoid, narrow or acute at the ends, arched or curved. Asterisks indicate the colonies’ formation by one end attached in mucilage. Autosporangia (arrowhead) and mother cell division into two daughter cells (arrow). (**g**,**h**): filaments and young cells (arrows) (see also [18]). (**i**,**j**): microscopic coccoid solitary cells. (**k**): coccoid young and mature cells, mother cell division into two daughter cells (asterisk). (**l**,**m**): young and mature cells arranged in dyad and tetrad aggregations (arrows). (**n**,**o**): mature vegetative cells form tight aggregations, colonies of randomly distributed cells (arrow), and aplanosporangia (asterisk). (**p**,**q**): young aplanosporia released from aplanosporangia (asterisk) (inset in (**q**)), mature vegetative cells form colonies (arrow), mucilage envelope is observable in solitary mature cells (arrowhead). All images are with DIC optics except (**a**,**j**,**l**,**m**,**o**). Bars, 10 μm.

**Figure 7:**
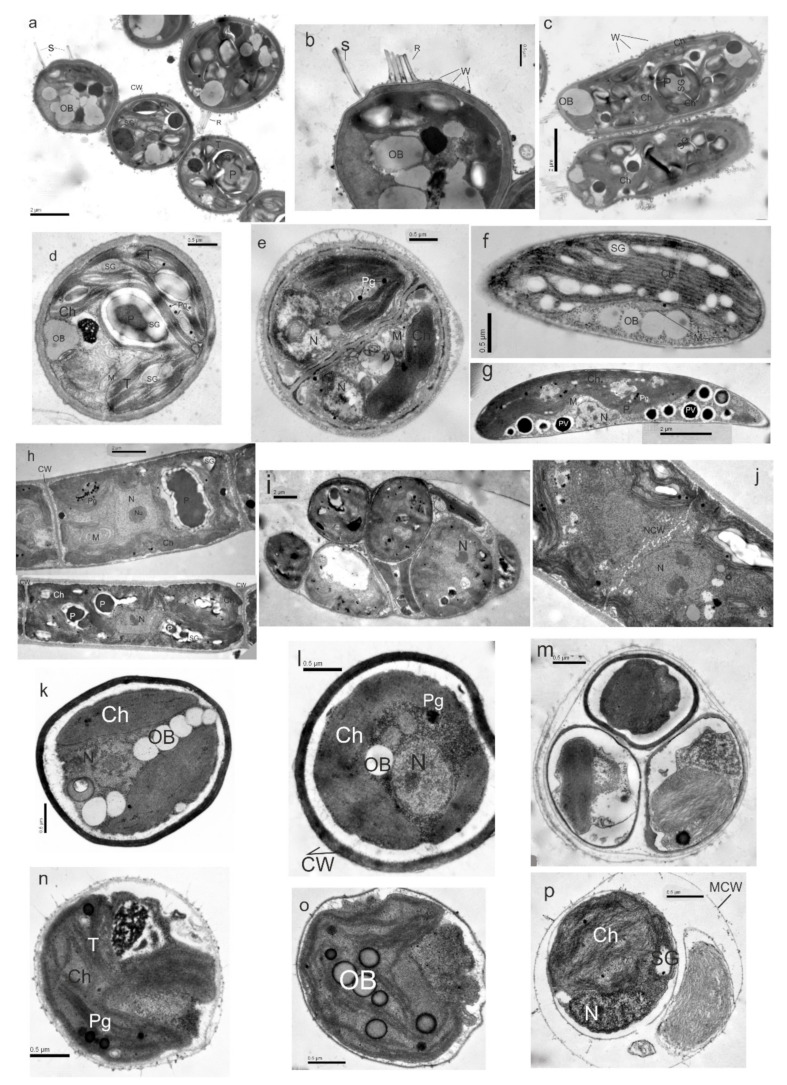
Electron micrographs of strains *Desmodesmus* sp. TAU-MAC 0910 (**a**–**c**), *Desmodesmus* sp. 1817 (**d**,**e**), *Monoraphidium* sp. 1210 (**f**,**g**), *Uronema trentonense* 0215 (**h**–**j**), *Ava limnothalassea* TAU-MAC 2217 (**k**–**m**), *Nomia picochloropsia* TAU-MAC 3617 (**n**–**p**); (**a**): four-celled coenobium with oil bodies and starch grains. (**b**): spines, ribs, and warts. (**c**): a central big pyrenoid surrounded by starch. (**d**): thylakoids of chloroplast arranged in bundles. One central pyrenoid surrounded by starch and accumulation of extra starch grains and lipid droplets (plastoglobuli, Pg) in the chloroplast. (**e**): a mother cell divided into two daughter cells. (**f**): oil bodies and starch grains are present in older cells. (**g**): polyphosphate vacuoles. (**h**): parietal and band-like chloroplast, containing more than one pyrenoid covered with starch grains. (**i**): autosporagium. (**j**): cytokinetic cell forming a new cell wall (NCW). (**k**): aging cell accumulating oil bodies. (**l**): mature cell, chloroplast without pyrenoid. (**m**): aplanosporangium. (**n**,**o**): mature cells that accumulate plastoglobuli. (**p**): aplanosporangium. Ch, chloroplast; CW, cell wall; M, mitochondrion; NCW, new cell wall N, nucleus; Nu, nucleolus; OB, oil body; Pg, plastoglobuli; P, pyrenoid; PV polyphosphate vacuole; R, ribs; S, spine; SG, starch grain; T, thylakoids; W, warts.

**Figure 8:**
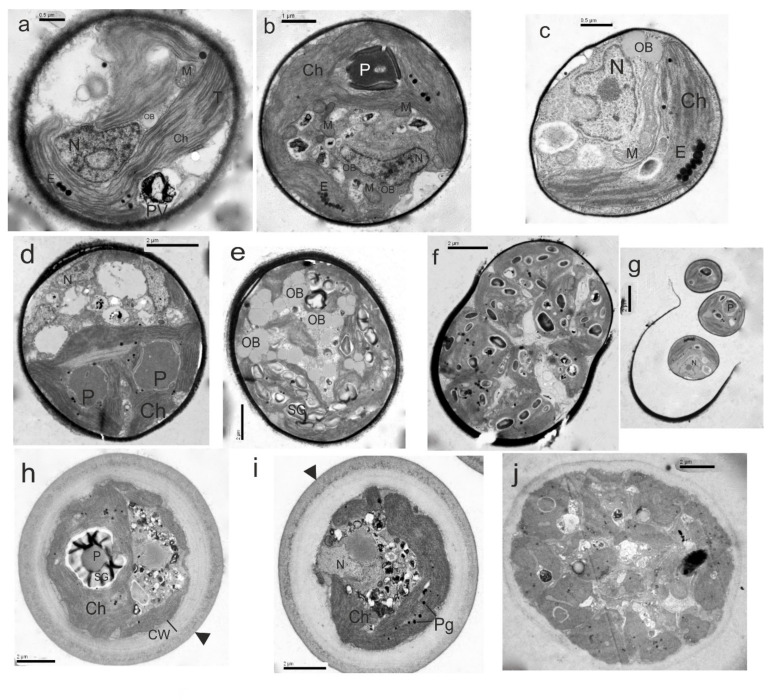
Electron micrographs of strains *Spongiosarcinopsis limneus* TAU-MAC 3310 (**a**,**b**), *Lilaea pamvotia* 3510 (**c**–**g**), *Akraea chliaropsychia* 0515 (**h**–**j**); (**a**,**b**): mature cells with eyespot, and pyrenoid surrounded by starch. (**c**: mature cell with eyespot. (**d**): cell with two pyrenoids in the chloroplast. (**e**): aging cell accumulating starch grains and oil bodies. (**f**): autosporangium with numerous autospores. (**g**): release of autospores. (**h**,**i**): mature cells covered with mucilaginous sheath (arrowheads). (**j**): autosporangium with numerous autospores. Ch, chloroplast; CW, cell wall; E, eyespot; M, mitochondrion; N, nucleus; OB, oil body; P, pyrenoid; Pg, plastoglobuli; PV polyphosphate vacuole; SG, starch grain; T, thylakoids.

In this study, eighteen photosynthetic microorganism strains belonging to Chlorophyta (Table 1), isolated from different aquatic environments, were characterized. A comparative analysis combining molecular (Figure 1, Figure 2, Figure 3 and Figure 4), morphological (Figure 5 and Figure 6) ultrastructural (Figure 7 and Figure 8) and ecological data led to the description of four new genera and five new species, belonging to the classes Chlorophyceae and Trebouxiophyceae (see Section 3.5).

Figure 1, Figure 2, Figure 3 and Figure 4 show the phylogenetic relationships of Chlorophyta TAU-MAC strains along with taxa of representative families belonging to the investigated orders, based on robust 18S rRNA gene, ITS region, and *rbcL* gene sequences analyses. Twelve strains belong to Sphaeropleales, two to Chlorellales, three to Chlamydomonadales, and one to Chaetophorales order (Figure 1, Figure 2, Figure 3 and Figure 4). Under light microscopy five morphotypes were recognized; ten strains corresponded to *Desmodesmus*-like species, two were coccoid, one filamentous, one crescent-like, one spherical to ovoid, and tree spherical (Figure 5 and Figure 6) (Table S2).

### 3.1. Sphaeropleales

Most of the TAU-MAC strains (0910, 1010, 1210, 0415, 1817, 1917, 2017, 2117, 2517, 2617, 2717, and 3917) are representatives of Sphaeropleales and were placed within the genera *Desmodesmus*, *Monoraphidium*, and *Asterarcys*. All those strains except TAU-MAC *Monoraphidium* sp. 1210 and *Asterarcys quadricellulare* 3917 were placed into *Desmodesmus*, one of the most plenteous genera in freshwater ecosystems worldwide [56]. The polymorphism they show is responsible for their tolerance and cosmopolitan occurrence in a wide range of environmental conditions [12,57]. The morphological variability of *Desmodesmus* due to nutrient availability and environmental signals has led to the description of more than 1300 species, many of which are invalid [44,56]. The great variability of the genus was clearly observed through microscopic observations in this study (Figure 5 and Figure 7). Based on ITS2 sequences analyses, An et al. [58] separated the genus from *Scenedesmus* sensu lato but the genus taxonomy still remains one long-standing issue in green microalgal systematics [59]. This was also obvious during our phylogenetic analyses, as we found a lot of misidentified sequences of *Scenedesmus* and *Desmodesmus*; they need taxonomic re-assignment, notably, *Scenedesmus* sequences which belong to *Desmodesmus,* and this is clearly depicted in our phylogenetic trees.

Based on the three genetic markers, TAU-MAC 0910, and 1010 formed distinct separate clades within *Desmodesmus* with high node support (Figure 1; clade 3, Figure 2a; clade 1, Figure 2b; clade 5). Direct 18S rRNA sequences comparison indicated as closest relatives a *D. insignis* sequence with 98.9% identity and an uncharacterized species of the genus with 97% similarity. After that, the percentage identity was very low, 92% with a *D. pannonicus* sequence (Table S3) isolated from China. Unfortunately, there was only one available sequence of *D. insignis*, both in nuclear and chloroplast-encoded gene sequences used in this study (18S rRNA, ITS region, *rbcL* gene). The closest relatives of ITS region sequences comparison included unidentified *Desmodesmus* species with high similarity of 99% and *rbcL* gene sequences had as closest relative a *D. pannonicus* sequence with 96.5% identity (Table S3). The strains displayed intermediate structures to *D. abundans* and *D. communis* but with shorter subpolar and lateral spines. Coenobia of two or four cells linearly arranged along their long axes were observed most frequently (Figure 5a,c and Figure 7a,c) but also eight-celled coenobia were observed (Figure 5b). Each cell included a single chloroplast with one big pyrenoid covered by starch plates (Figure 7a,c) while extra starch grains and oil bodies were found in cells of older cultures (Figure 7a–c). Cell wall ornaments such as spines, warts, and rosettes were observable from cell wall residues (Figure 5a) and under TEM (Figure 7b). Because of the extensive morphological variability of the genus, and overlapping between species, no differences in morphology or a special trait of the strains that could separate them from other species were observed.

The strains TAU-MAC 1817, 2017, and 2617 displayed distinct phylogenetic positions which were converged both in nuclear and chloroplast genes trees (Figure 1 and Figure 2); they formed together strongly supported and phylogenetically distinct separate clades among species of *Desmodesmus* in 18S rRNA (Figure 1; clade 6), ITS region (Figure 2a; clade 5) and *rbcL* gene trees (Figure 2b; clade 4). In the 18S rRNA tree, they clustered together into *Desmodesmus* clade forming a strongly supported, independent branch with two unidentified taxa of the genus (AY197627.1 and AY197638.1) for which, there was no available information (morphology, isolation, etc.) (Figure 1; clade 6). Similarly, based on ITS region phylogeny, they formed an independent branch with an unclassified representative of the genus (KT445862.1) isolated from water streams in Brazil (Figure 2a; clade 5). Direct 18S rRNA and ITS sequences comparisons revealed, as closest relatives of strains (Tables S3 and S4), unidentified *Desmodesmus* species, which were included in the same branches with our isolates. Based on *rbcL* gene phylogeny, isolates had closest relatives and were placed together in a branch, with two “*Acutodesmus deserticola*” (KT777973, KT777976) (currently regarded as a synonym of *Tetradesmus deserticola*) sequences (Table S5). However, the phylogenetic position of the real *Tetradesmus* clade (Figure 2b; clade 7) in the *rbcL* gene tree, is clearly distant from the clade where the strains were grouped [60,61], as well as in the 18S rRNA tree (Figure 1; clade 7). Additionally, *T. deserticola* is a terrestrial species [5], and the strains 1817, 2017, and 2617 were isolated from water. Therefore, our data suggest that the “*Tetradesmus*” clade inside *Desmodesmus* genus in the *rbcL* gene tree, was formed by two misidentified *Desmodesmus* species (Figure 2b; clade 4). The strains’ morphology is simple: small spherical green cells (Figure 5d–g), a single chloroplast with a big pyrenoid covered by starch (Figure 7d); propagation by aplanosporogenesis or cell division of a mother cell into two daughter cells (Figure 7e). Two-celled and four-celled coenobia were sometimes observed but solitary cells were predominant. This comes in contrast to the fact that most species of the genus are organized in coenobia [62]. No spines, flagellae, or cell wall ornaments were observed (Figure 5d–g and Figure 7d,e). Despite the evidence we have for both *Desmodesmus* strains, it is difficult to come to a safe conclusion regarding their *taxonomic treatment*. Genus *Desmodesmus* includes many misidentified species and has a complex history, thus further research is needed to conclude their taxonomy.

The strain TAU-MAC 0415 falls inside *Desmodesmus subspicatus* clade with strong support, such as *D. subspicatus* TAU-MAC 2810. Phylogenetic relationships of 0415 were supported by three molecular markers (Figure 1; clade 1, Figure 2a; clade 2, Figure 2b; clade 3). Morphological taxonomy was congruent to the molecular classification; TAU-MAC 0415 exhibited the morphological structure of *D. subspicatus*; ovoid and elongated cells, 5–13 µm long and 3–7 µm wide and chloroplast with one large pyrenoid. The four-celled coenobia were predominant, followed by two-celled coenobia and solitary cells (Figure 5h). Lateral spines of outer cells were longer than those of inner cells (Figure 5h) and reproduction was asexual by aplanosporogenesis or cell division of a mother cell into two daughter cells (Figure 5h) [45]. Furthermore, comparative microscopic observations with *D. subspicatus* TAU-MAC 2810 showed high morphological similarity, specifically in the presence of lateral spines both in outer and inner cells [18] (Figure 5h).

The strains TAU-MAC 1917 and 2517 were grouped together with unclassified species of the genus *Desmodesmus* in the 18S rRNA tree having high sequence similarity >99% (Figure 1; clade 2, Table S3). In *rbcL* gene phylogeny, strains were placed in a clade with unclassified species and a *D. multivariabilis* sequence (shared 97.4% similarity, Table S5) (Figure 2b; clade 2). Based on ITS region phylogeny, strains were placed in *D. multivariabilis* clade sharing > 98% sequence similarity (Figure 2a; clade 3), reaching 100% in the ITS2 region (Table S4). The sequence comparison among 18S rRNA of TAU-MAC strains and *D. multivariabilis* was not possible due to the lack of available sequences. The species has a significant morphological range as its name also indicates [63,64] with strains TAU-MAC 1917 and 2517 displaying some of the characteristics such as the ovoid and elongated cells, the formation of two, four, or eight-celled coenobia surrounded by mucilage (Figure 5i,j). Unicells were more common but also the spineless coenobia of two or four cells were observed (Figure 5i). To the best of our knowledge for both *D. subspicatus* TAU-MAC 0415 and *D. multivariabilis* TAU-MAC 1917 and 2517, this is the first report of their occurrence in a hot spring environment.

Based on 18S rRNA and ITS region phylogenies TAU-MAC 2117 and 2717 were placed into *D. abundans* branch together with strains *D. abundans* TAU-MAC 0810 and 3110 (Figure 1; clade 4, Figure 2a; clade 4), along with the reference strain of the species (*D. abundans* CCAP 258/211-213). The *D. abundans* clade in the *rbcL* gene phylogeny contained all TAU-MAC *D. abundans* sequences (0810, 3110, 2117, 2717) together with only one unidentified Chlorophyta sequence (Figure 2b; clade 1). *D. abundans* TAU-MAC 2117 and 2717 exhibited the morphological features of the species; cells spherical to ovoid and elongated, single, spineless, cells were dominant, while the rare four-celled coenobia were always spined with one lateral and two polar spines in the outer cells (Figure 5k,l) [43]. Furthermore, through morphological comparison, strains were similar to *D. abundans* TAU-MAC 0810 and 3110 [18]. The species, like many others of the genus, exhibits extensive morphotype variability [44], also observed in our cultures. The species occurs in different freshwater ecosystems worldwide [43,65] but this is the first report of its isolation from an aquatic environment up to 40 °C (Agkistro Hot Springs).

Both molecular and morphological data support the identification of TAU-MAC 3917 as *Asterarcys quadricellulare*. In 18S rRNA and ITS region phylogenetic trees, the isolate was clustered with high support, into *Asterarcys quadricellulare* clade (Figure 1; clade 8, Figure 2a; clade 6) including the reference strain of the species (*A. quadricellulare* Comas 77/75). Their closest relative strains were isolated from water (Cuba) and soil (Egypt, China) [66,67,68] (Tables S3 and S4) while *A. quadricellulare* TAU-MAC 3917 was isolated from a pond inside AUTH campus with stagnant water. *A. quadricellulare* TAU-MAC 3917 displayed irregularly spherical big cells, arranged in coenobia of randomly distributed cells which were surrounded by mucilage envelope (Figure 6a–c), similar features to *A. quadricellulare* [67]. Solitary cells were observed most frequently, while their reproduction was asexual by autospores (Figure 6c). *A. quadricellulare* TAU-MAC 3917 is capable to accumulate pigments in older cultures (changing the color of algal mass from green to brick-red) as was observed under light microscopy (Figure 6b) and cytoplasmic lipids as was observed under TEM (data not shown). The genus *Asterarcys* was considered to be part of *Coelastrella* sensu lato clade [69], a hypothesis that was not supported by the morphology of *Asterarcys* which is clearly distinct morphology compared to *Coelastrella* [70]. According to Wang et al. [70] phylogeny based on the *tuf*A chloroplast gene supported the separation of the genera as *Asterarcys* formed a separate branch outside of *Coelastrella* clade and that was also clear in this study. The genus is planktic occurring in eutrophic lakes and ponds in tropical Asia, Central America, and the Caribbean [67,71] likewise the strain TAU-MAC 3917, which was isolated form a eutrophic pond with stagnant water inside AUTH campus.

TAU-MAC 1210 was placed into the *Monoraphidium* clade both in 18S rRNA and *rbcL* gene phylogeny (Figure 1; clade 9, Figure 2b; clade 8). Direct sequences comparison revealed high similarity (>99%) among 18S rRNA different species sequences of the genus (Table S3). Among ITS region sequences of the clade, the percentage identity is very low <93% (Table S4) whilst the strain was placed alone in a branch between the clades of close genera *Ankistrodesmus* and *Messastrum* (Figure 2a; clade 7). Phylogeny-based in the ITS region did not resolve the relationships among Selenastraceae lineage due to the lack of deposited ITS sequences and the low support of some internal branches. In the *rbcL* gene phylogenetic tree, TAU-MAC 1210 fell inside the *Monoraphidium* clade, in a separate branch (Figure 2b; clade 8), sharing <97% sequence similarity with *Monoraphidium* species (Table S6). Lots of taxonomic advances have proceeded in the family since its description in 1903 [72]. Even if the monophyly of Selenastraceae was elucidated [73,74,75] the necessity for genera revision within the family is pointed out, since molecular data do not support morphological identification and disjunction [74,75]. TAU-MAC 1210 exhibited features of the genus *Monoraphidium*; unicellular microalga without mucilage, fusiform, curved to sigmoid, narrow or acute at the ends (Figure 6d–f) [76], including one parietal chloroplast with an embedded naked pyrenoid, without starch cover (Figure 7f,g). The reproduction by the autospores was asexual (Figure 6f) as is known for the genus [73,77]. Colony formation was scarcely observed by one end attached in mucilage (Figure 6e), a distinguishing trait of *Monoraphidium* [77]. Representatives of Selenastraceae are included among the most common members of inland waters phytoplankton communities and they have served as biological indicators of unhealthy conditions in natural ecosystems [72]. Among members of the family, morphological diversity is remarkable; coccoid and spherical to elongated and fusiform, sickle-shaped, lunate, spirally curved, with sharp or rounded ends, unicells to colonial forms [73,75,78]. The phylogenetic position of the isolate *Monoraphidium* sp. TAU-MAC 1210 deserves further research to conclude to certain identification. The strain was isolated from the hypertrophic, Lake Pamvotis where the formation of heavy toxic cyanobacterial blooms is frequent [79].

### 3.2. Chaetophorales

The strain TAU-MAC 0215 was grouped into *Uronema* clade in order Chaetophorales based on 18S rRNA and ITS region phylogeny (Figure 1; clade 11, Figure 4a; clade 7). The strain shared >99% 18S rRNA sequence similarity (Table S7) with all *Uronema* species included in the clade 11. Based on ITS sequence comparison, the closest relative (98.8% similarity) was an unidentified *Uronema* sequence (Table S8). ITS2 region sequence similarities among the only available deposited ITS region sequence of the species *Uronema trentonense* (HF920659.1) and the isolate 0215 was extremely high >99.5%. Furthermore, the strain displayed all the morphological traits of *U. trentonense* which was isolated from soil [39]; filaments uniseriate, unbranched, attached to the substratum by holdfast, bearing a pointed apical cell at the free end, and indefinite in length (Figure 6g,h and [18]). Under TEM the strain exhibited one parietal chloroplast per cell, with more than one pyrenoid covered with starch (Figure 7h). Asexual reproduction took place by aplanosporogenesis or zoosporogenesis (Figure 7i). *U. trentonense* TAU-MAC 0215 was isolated as a benthic species, from an aquatic environment with a temperature up to 40 °C and, to the best of our knowledge, this is the first report of the presence of *Uronema* in such conditions. The rbcL gene sequence of the strain could not be amplified. Species of the genus appeared as benthic algae in mesotrophic/eutrophic ecosystems while they are common in both terrestrial and aquatic environments [39,80].

### 3.3. Chlorellales

Our polyphasic analysis of the strains TAU-MAC 2217 and 3617 isolated from the surface water of Kalochori Lagoon supports the delineation of two new genera within Trebouxiophyceae. TAU-MAC 2217 was clustered along with sequences denoted as “*Nannochloris*” (Table S9) in 18S rRNA phylogeny, isolated from several saline environments, into a strongly supported and clearly separate clade, with a sequence similarity >99% (Figure 1; clade 13). However, this clade is phylogenetically distinct from *Nannochloris* clade (clade 15) which is considered to include true species of the genus [81,82,83]. *Nannochloris* originally described by Naumann [84] included two species *N. bacillaris* Naumann and *N. cocoides* Naumann, but no holotype or lectotype specimens were given and molecular data were not provided for type strains [85]. TAU-MAC 2217 ITS and *rbcL* gene phylogenies support the delineation of a new genus, since the strain falls outside of the “true” *Nannochloris* clades (Figure 3a,b; clade 3); it was placed alone in a distinct clade into Chlorellaceae, clearly separated from the phylogenetically close genera *Chlorella*, *Auxenochlorella*, *Marvania*, and *Nannochloris*. Its closest relatives with very low similarity are *Chlorella sorokiniana* (85%) and a *Nannochloris* (91%) sequence, respectively (Tables S10 and S11). The newly described genus and species *Laetitia sardoa* [86] has 96.7% 18S rRNA sequence identity and 90.26% *rbcL* sequence identity with TAU-MAC strain. Under light and transmission electron microscopy the cells did not exhibit variations from other *Nannochloris*-like morphotypes as they are considered to be morphologically the simplest phototrophic eukaryotes (Figure 6i,j and Figure 7k–m) [85]. Thus, a new genus of Chlorellales *Ava* gen. nov. is proposed (see Section 3.5). Its type species *Ava limnothalassea* exhibited solitary cells, green, spheroidal, and microscopic (Figure 6i,j), containing a cup-shaped chloroplast without pyrenoid while aging cells accumulated oil bodies and plastoglobuli (Figure 7k,l). Their reproduction was by aplanospores. (Figure 7m).

The second isolate belonging to Chlorellales and isolated from the Kalochori Lagoon, TAU-MAC 3617, was placed alone in a separate clade in 18S rRNA phylogeny (Figure 1; clade 14), distinct from the sister *Ava*-“*Nannochloris*” clade (Figure 1; clade 15) and close to the genera *Chlorella*, *Marvania*, *Nannochloris,* and *Auxenochlorella* (Chlorellaceae family). In the *rbcL* gene tree, it formed a well-supported clade along with a “*Picomonas*” sequence which is the closest relative with high similarity >99% (Figure 3b; clade 4). Nevertheless, the genus *Picomonas* is a member of the Phylum Picozoa and lacks plastids [87]. The “*Picomonas*” sequence, isolated from coastal Arabian Gulf waters, is referred to as *Picochloris* in the respective study [88] but no details about its identification were given. A direct comparison of *rbcL* gene sequence similarities among the TAU-MAC 3617 strain and *Picochlorum* representatives showed <93% sequence identity (Table S11). *Laetitia sardoa* has a clearly distinct phylogenetic position in both 18S rRNA and *rbcL* trees with TAU-MAC strain. TAU-MAC 3617 demonstrated the typical morphotype of the genus *Picochlorum* [85] except for its bigger cell size. The cells of TAU-MAC 3617 were spherical to oval (Figure 6k), with one lateral cup-shaped chloroplast without pyrenoid (Figure 7n). Aging cells accumulated large plastoglobuli (Figure 7o) and reproduced by aplanospores (Figure 7p). The ITS sequence could not be retrieved. Summarizing this evidence, the new genus of Chlorellales *Nomia* gen. nov. and its type species *Nomia picochloropsia* are proposed (see Section 3.5).

### 3.4. Chlamydomonadales

Three TAU-MAC strains 3310, 3510, and 0515 are representatives of Chlamydomonadales the largest group of Chlorophyceae the systematics of which are complicated and constantly reviewed [1]. As summarized by Leliaert et al. [1], this order includes, except for a large number of Chlamydomonadales taxa, taxa placed earlier in Tetrasporales, Volvocales, Chlorococcales, Dunaliellales, and Chaetophorales.

TAU-MAC 3310 was clustered into *Spongiosarcinopsis terrestris* clade both in 18S rRNA and *rbcL* gene phylogeny (Figure 1; clade 18, Figure 4b; clade 3), a novel, recently described terrestrial green algae genus and species isolated from soil coming from a gray forest in Russia [7]. The *rbcL* gene sequence similarity among TAU-MAC 3310 and deposited *S. terrestris* sequence is <97% (Table S14). In ITS the phylogenetic tree TAU-MAC 3310 was grouped with a “*Balticola*” sequence (MH068690) isolated from freshwater in India, sharing 96% similarity (Figure 4a; clade 4) (Table S13). Nevertheless, the clade that includes the true *Balticola* sequences is phylogenetically distinct from the TAU-MAC 3310 clade (Figure 4a; clade 3). No further information about this “*Balticola*” strain was available to compare with the TAU-MAC isolate. The complete ITS region sequence of *S. terrestris* was not available to be included in the analysis. However, direct comparison of the available ITS2 region sequences among *S. terrestris* and TAU-MAC 3310, revealed a very low similarity (81.7%). The strain exhibited the morphological characteristics of the new genus mainly in coenobia formation that were organized into dyads, tetrads, or packets resulting from desmoschisis; ellipsoidal to spherical solitary young cells becoming ovoid to irregular by aging cells (Figure 6l,m and Figure 8a,b). Although TAU-MAC 3310 cultures were maintained for months in order to observe the accumulation of secondary carotenoids by changes in the color of algal mass, as was detected by Temraleeva et al. [7] in older cultures, this feature was not observed. Concluding, the low similarity of the ITS2 region and *rbcL* gene sequences, the different niches (aquatic vs terrestrial), and the differences in conditions of aged cells, suggest that TAU-MAC 3310 belongs to another *Spongiosarcinopsis* species. Thus, a new *Spongiosarcinopsis* species is proposed herein, *S. limneus* (see Section 3.5).

Strain TAU-MAC 3510 formed a well-supported clade with a “*Chlorococcum*” sequence (KF791546.1) in a 18S rRNA tree (Figure 1; clade 17), that was isolated from an extreme saline-alkali soil sample in China [89]. Nevertheless, the *“Chlorococcum*” clade was placed in all of the three trees, in phylogenetically distinct clades from the TAU-MAC 3510 branches (Figure 1; clade 19, Figure 4a; clade 1, Figure 4b; clade 4). Chlamydomonadales has a complex taxonomic history, including several genera that are polyphyletic and may need revision [1]. *Chlorococcum* is considered polyphyletic and efforts have been made to reassess misidentified species and elucidate the phylogenetic relationships among them [90,91]. Nevertheless, there are many not revised sequences deposited in Genbank as was noticed also during this study. In the ITS region and *rbcL* gene phylogenetic trees, the isolate was placed alone in separate branches (Figure 1; clade 17, Figure 4a; clade 5, Figure 4b; clade 1) distinct from the close genera *Protoshiphon*, *Tetracystis*, *Spongiosarcinopsis*, and *Chlorococcum*. ITS region sequence showed <85% similarity with the close genus *Tetracystis* (Table S13), while the *rbcL* gene sequence showed <95% with *Protosiphon* (Table S14). TAU-MAC 3510 exhibits spherical to irregular vegetative cells with the ability to form cell aggregates and sometimes colonies of randomly distributed cells (Figure 6n,o). Cells contained cup-shaped chloroplast (Figure 8c–e), sometimes with eyespot (Figure 8c) and pyrenoids surrounded by starch plates (Figure 8d) and the reproduction was asexual by aplanospores (Figure 8f,g). Even if TAU-MAC 3510 shared some morphological features with *Chlorococcum* such as cell forms and mode of reproduction [90,91], they appear to be distant in molecular phylogeny. In summary, we propose a new Chlamydomonadales genus, *Lilaea,* and its type species *L. pamvotia* (see Section 3.5), largely based on molecular distance and low sequence similarity with its close genera.

TAU-MAC 0515 formed a highly supported clade within Chlamydomonadales in the 18S rRNA tree, with uncharacterized sequences together with two “*Chlamydopodium*” and one “*Chlamydomonas*” sequence (Figure 1; clade 21). Direct sequence comparison revealed >99% similarity between them (Table S12). However, this clade was placed distinctly from the true *Chlamydopodium* (Figure 1; clade 20) and *Chlamydomonas* (Figure 1; clade 16) clades that are placed in separate branches including the sequences with accession numbers M63001.1, AB983625.1 and AB511834.1, AB511839.1 respectively [89,91,92]. The broad number of misidentified Chlamydomonadales deposited sequences, is also clear here. The well-supported relationships of 0515 and their relatives, were also found in the ITS region and *rbcL* gene phylogeny. Both genera *Chlorococcum* (Figure 4a; clade 1, Figure 4b; clade 4) and *Chlamydomonas* (Figure 4a; clade 6, Figure 4b; clade 5) were placed in clearly separate branches from TAU-MAC 0515. In ITS region phylogeny, the strain was clustered with an unidentified Chlamydomonadaceae and a “*Chlamydomonas*” sequence sharing together >99% similarity (Figure 4a; clade 2) (Table S13). In *rbcL* gene phylogeny, TAU-MAC 0515 falls inside a highly supported clade with the closest relative, a *Chlamydopodium* sequence sharing a low sequence identity of 93% (Figure 4b; clade 2) (Table S14). The cells of TAU-MAC 0515 were spherical to irregular, covered with a mucilaginous envelope, with the ability to form cell aggregates (Figure 6p,q). TAU-MAC 0515 shared some morphological features with the genus *Chlamydopodium* except for the mucilaginous basal pad *Chlamydopodium* uses to attach to the substratum [93]. Moreover, the strain exhibited morphological characters of the sister genus *Chlorococcum*, such as chloroplast structure and the ability to form cell aggregates [91,92] (see ultrastructure in Figure 8h–j) but differed due to its inability to form colonies. Both sister genera have flagellate cell forms which in TAU-MAC 0515 could not be detected through microscopy. Furthermore, TAU-MAC 0515 was isolated from an aquatic environment with a temperature > 40 °C (Agkistro Hot Springs). Thus, we propose herein a new genus inside Chlamydomonadales, *Akraea,* and its type species *A. chliaropsychia* (see Section 3.5).

### 3.5. Taxonomic Descriptions

***Ava* Lortou and Gkelis gen. nov. (Figure 1, Figure 3, Figure 6i,j and Figure 7k–m; Tables S2 and S9–S11)**

Description: Cells are green, solitary ovoid to spheroidal, microscopic, 2–7 μm in diameter, growing in water, either saline or fresh. One nucleus, chloroplast single, cup-shaped, or parietal lacking a pyrenoid. Asexual reproduction by aplanosporogenesis, two-four aplanospores per sporangium.

Etymology: From the Greek Άβα (Ava), one of the Naiads; in Greek mythology, the naiads are a type of female spirit, or nymph presiding over lakes, wells, springs, streams, brooks, and other bodies of freshwater.

Type species: *Ava limnothalassea* TAU-MAC 2217 Lortou and Gkelis sp. nov.

Occurrence: marine, freshwater, planktic.

***Ava limnothalassea* Lortou and Gkelis sp. nov. (Figure 1, Figure 3, Figure 6**i**,j and Figure 7k–m, Tables S2 and S9–S11)**

Description: Cells are green, solitary spherical or oval, microscopic, 2–7 μm in diameter, growing in water, either saline or fresh. One nucleus, chloroplast single, cup-shaped, or parietal without a pyrenoid. Young cells are ellipsoidal, becoming spherical at maturity. Aging cells accumulate high levels of lipid. Asexual reproduction by aplanosporogenesis, two-four aplanospores per sporangium. Sexual reproduction unknown.

Etymology: From the Greek λιμνοθάλασσα (limnothalassa) = lagoon, estuary

Holotype: TAU-A01026 Thessaloniki Aristotle University (TAU) Herbarium, Thessaloniki, Greece (dried biomass and fresh sample maintained in formaldehyde, collected by U.L on 19 January 2022).

Reference strain: TAU-MAC 2217 Thessaloniki Aristotle University Microalgae and Cyanobacteria (TAU-MAC) culture collection.

Type locality: This taxon occurred as planktic in water of Kalochori Lagoon (40°27′53″ N, 22**°**51′48″ E) and collected on 1 November 2017 from a surface layer (0–0.5 m) of inshore sites.

GenBank accession numbers: OK641936.1, OK642365.1, OK626429.1

***Nomia* Lortou and Gkelis gen. nov. (** **Figure 1, Figure 3, Figure 6k and Figure 7** **n–p, Tables S2 and S9–S11)**

Description: Cells are green, solitary spherical or oval, microscopic, 2–8 μm in diameter, growing in water, either saline or fresh. One nucleus, chloroplast single, lateral cup-shaped or discoid without a pyrenoid. Cell wall thin with bristle-like hair on the surface. Asexual reproduction by autosporulation.

Etymology: From the Greek Νόμια (Nomia), one of the Naiads; in Greek mythology, the naiads are a type of female spirit, or nymph presiding over lakes, wells, springs, streams, brooks, and other bodies of freshwater.

Type species: *Nomia picochloropsia* TAU-MAC 3617 Lortou and Gkelis sp. nov.

Occurrence: marine, freshwater, planktic.

***Nomia picochloropsia* Lortou and Gkelis sp. nov. (** **Figure 1** **, Figure 3** **, Figure 6** **k and Figure 7** **n–p, Tables S2 and S9–S11)**

Description: Cells are green, solitary, ovoid to spheroidal, microscopic, 2–9 μm in diameter, growing in water, either saline or fresh. One nucleus, chloroplast single, lateral cup-shaped without a pyrenoid. Starch grains sometimes present. Aging cells accumulate high levels of lipid. Asexual reproduction by autosporulation. Sexual reproduction unknown.

Etymology: picochlorum (L.) = genus *Picochlorum*; -opsis (Gr.) = looking like, alike; morphologically similar to *Picochlorum*.

Holotype: TAU-A01027 Thessaloniki Aristotle University (TAU) Herbarium, Thessaloniki, Greece (dried biomass and fresh sample maintained in formaldehyde, collected by U.L on 19 January 2022).

Reference strain: TAU-MAC 3617 Thessaloniki Aristotle University Microalgae and Cyanobacteria (TAU-MAC) culture collection.

Type locality: This taxon occurred as planktic in water of Kalochori Lagoon (40°27′53″ N, 22°51′48″ E) and collected on 1 November 2017 from a surface layer (0–0.5 m) of inshore sites.

GenBank accession numbers: OK641940.1, OK626437.1.

***Spongiosarcinospis limneus* Lortou and Gkelis sp. nov. (Figure 1, Figure 4, Figure 6l,m and Figure 8a,b, Tables S2 and S12–S14)**

Description: Ellipsoidal to spherical solitary young cells 4–9 µm in diameter. Under TEM, young cells contain one nucleus, one parietal chloroplast with one big pyrenoid covered with starch envelope. Mature cells ovoid to irregular in shape, >20 µm in dimension, they organized into dyads, tetrads, or packets resulting from desmoschisis. Chloroplast of mature cells is spongy, possessing one pyrenoid surrounded by a sheath of starch plates. Eyespots present. Asexual reproduction is possible by desmoschisis or zoospores and aplanospores.

Etymology: The specific epithet refers to the habitat of the isolate. From the Greek λιμναίος (limnaeos) = referring to lake. 

Holotype: TAU-A01028 Thessaloniki Aristotle University (TAU) Herbarium, Thessaloniki, Greece (dried biomass and fresh sample maintained in formaldehyde, collected by U.L. on 19 January 2022).

Reference strain: TAU-MAC 3310 Thessaloniki Aristotle University Microalgae and Cyanobacteria (TAU-MAC) culture collection.

Type locality: This taxon occurred as planktic in water column of Lake Doirani (41°18′56″ N, 22°45′37″ E) and collected on 21 August 2010 from a surface layer (0–0.5 m) of inshore sites.

GenBank accession numbers: MK496896.1, MK496929.1, OK626435.1.

***Lilaea* Lortou and Gkelis gen. nov. (** **Figure 1** **, Figure 4** **, Figure 6** **n,o and Figure 8** **c–g, Tables S2 and S12–S14)**

Description: Solitary vegetative cells spherical to irregular form, 7–16 μm in diameter with the ability to form cell aggregates without mucilage. Sometimes arranged in colonies of randomly distributed cells. Chloroplast cup-shaped to reticulate with eyespot and one or several pyrenoids surrounded by starch plates. Single nucleus or multiple nuclei directly before reproduction by aplanospores. Starch and plastoglobuli in chloroplasts and cytoplasmic oil bodies accumulate in aged cultures. Thylakoids in bundles of different sizes and thicknesses. Asexual reproduction by aplanospores and may have zoospores.

Etymology: From the Greek Λιλαία (Lilaea), one of the Naiads; in Greek mythology, the naiads are a type of female spirit, or nymph, presiding over lakes, wells, springs, streams, brooks, and other bodies of freshwater.

Type species: *Lilaea pamvotia* TAU-MAC 3510 Lortou U. and Gkelis S. sp. nov.

Occurrence: freshwater, planktic.

***Lilaea pamvotia* Lortou and Gkelis sp. nov. (** **Figure 1** **, Figure 4** **, Figure 6** **n,o and Figure 8** **c–g, Tables S2 and S12–S14)**

Description: Vegetative cells spherical to irregular form, 7–16 μm in dimension, form cell aggregates without mucilage and sometimes arranged in colonies of randomly distributed cells. Chloroplast cup-shaped to reticulate, thylakoids organized in bundles of various thickness, sometimes with eyespot and one or several pyrenoids surrounded by starch plates. Single nucleus. A lot of starch and plastoglobuli (lipid droplets) in chloroplasts and cytoplasmic oil bodies accumulate in aged cultures. Thylakoids in bundles of different sizes and thicknesses. Asexual reproduction by aplanospores.

Etymology: From Gr. Παμβώτις (Pamvotis) = the Lake Pamvotis in NW, Greece, type locality of the species.

Holotype: TAU-A01029 Thessaloniki Aristotle University (TAU) Herbarium, Thessaloniki, Greece (dried biomass and fresh sample maintained in formaldehyde, collected by U.L. on 19 January 2022). (Figure 6n,o and Figure 8c–g).

Reference strain: TAU-MAC 3510 Thessaloniki Aristotle University Microalgae and Cyanobacteria (TAU-MAC) culture collection.

Type locality: This taxon occurred as planktic in water column of Lake Pamvotis (39°40′51″ N, 20°50′30″ E) and collected on 1 November 2010 from a surface layer (0–0.5 m) of inshore sites.

GenBank accession numbers: MK496896.1, MK496929.1, OK626435.1.

***Akraea* Lortou and Gkelis gen. nov. (** **Figure 1** **, Figure 4** **, Figure 6** **p,q and Figure 8** **h–j, Tables S2 and S12–S14)**

Description: Solitary vegetative cells spherical to irregular form, 9–18 μm in diameter, covered with mucilaginous sheath and sometimes arranged in colonies of randomly distributed cells. Chloroplast cup-shaped to reticulate with eyespot and one big pyrenoid surrounded by starch plates. Single nucleus. Starch and lipid droplets accumulate in chloroplasts in aged cultures. Thylakoids in bundles of different sizes and thicknesses. Asexual reproduction by aplanospores and may have zoospores.

Etymology: From the Greek Aκραία (Akraea), one of the Naiads; in Greek mythology, the naiads are a type of female spirit, or nymph, presiding over lakes, wells, springs, streams, brooks, and other bodies of freshwater.

Type species: *Akraea chliaropsychia* TAU-MAC 0515 Lortou U. and Gkelis S. sp. nov.

Occurrence: in water, hot springs.

***Akraea chliaropsychia* Lortou and Gkelis sp. nov. (** **Figure 1** **, Figure 4** **, Figure 6** **p,q and Figure 8** **h–j, Tables S2 and S12–S14)**

Description: Vegetative cells spherical to irregular form, 9–22 μm in diameter, covered with thick mucilaginous sheath and sometimes arranged in colonies of randomly distributed cells. Chloroplast parietal to reticulate with eyespot and one big pyrenoid surrounded by starch plates. Single nucleus. Starch and plastoglobuli accumulate in chloroplasts in aged cultures. Thylakoids in bundles of different sizes and thicknesses. Asexual reproduction by aplanospores.

Etymology: From the Greek χλιαροψύχιον (chliaropsychion) = sub-thermal, the space before entering the warm Byzantine baths, the tepidarium.

Holotype: TAU-A01030 Thessaloniki Aristotle University (TAU) Herbarium, Thessaloniki, Greece (dried biomass and fresh sample maintained in formaldehyde, collected by U.L. on 19 January 2022).

Reference strain: TAU-MAC 0515 Thessaloniki Aristotle University Microalgae and Cyanobacteria (TAU-MAC) culture collection.

Type locality: This taxon occurred as planktic in water column of Agkistro Hot Springs (41°22′04″ N, 23°25′40″ E) and collected on 20 October 2015 from a surface layer (0–0.5 m).

GenBank accession numbers: OK641928.1, OK642360.1, OK626421.1

## 4. Conclusions

The importance of microalgae diversity knowledge is explicit for monitoring the ecological status of aquatic environments and their intra-ecosystem relationships. Combining molecular, morphological, and ecological data, this study proposed the designation of novel lineages within photosynthetic microorganisms. Mediterranean ecoregion has an excessive [16,31] level of diversity and endemism of microalgae species, however, is an unexplored area, as emphasized by the number of novel taxa (nine) characterized in this study. The results derived from this study revealed novel diversity among Chlorophyta and enlightened the phylogenetic relationships within the phylum. Furthermore, our findings and the problems we encountered during this study, indicate the necessity of applying polyphasic culture-dependent approaches in systematic research to assure microalgae identification and taxonomy.

## Figures and Tables

**Table 1:** Green algae strains investigated in this study, their origin, and habitat. Novel taxa described in this study are indicated in bold.

Strain	Origin	Geographic Coordinates		Habitat	Collection Date
		(N)	(E)		
*Desmodesmus* sp. TAU-MAC 1817	Agkistro Hot Springs	41°22′04″	23°25′40″	planktic	17 October 2017
*Desmodesmus multivariabilis* TAU-MAC 1917				planktic	
*Desmodesmus* sp. TAU-MAC 2017				planktic	
*Desmodesmus abundans* TAU-MAC 2117				planktic	
*Desmodesmus multivariabilis* TAU-MAC 2517				planktic	
*Desmodesmus* sp. TAU-MAC 2617				planktic	
*Desmodesmus abundans* TAU-MAC 2717				planktic	
***Ava limnothalassea* TAU-MAC 2217**	Kalochori Lagoon	40°27′53″	22°51′48″	planktic	1 November 2017
***Nomia picochloropsia* TAU-MAC 3617**				planktic	
*Asterarcys quadricellulare* TAU-MAC 3917	AUTH water pond	40°37′58″	22°57′21″	planktic	1 November 2017
*Uronema trentonense* TAU-MAC 0215	Agkistro Hot Springs	41°22′04″	23°25′40″	benthic	20 October 2015
*Desmodesmus subspicatus* TAU-MAC 0415				planktic	
***Akraea chliaropsychia* TAU-MAC 0515**				planktic	
***Spongiosarcinopsis limneus* TAU-MAC 3310**	Lake Doirani	41°18′56″	22°45′37″	planktic	21 August 2010
*Desmodesmus* sp. TAU-MAC 1010	Lake Koronia	40°42′04″	23°08′17″	planktic	30 August 2010
***Lilaea pamvotia* TAU-MAC 3510**	Lake Pamvotis	39°40′51″	20°50′30″	planktic	1 November 2010
*Monoraphidium* sp. TAU-MAC 1210				planktic	
*Desmodesmus* sp. TAU-MAC 0910	Lake Volvi	40°40′37″	23°33′10″	planktic	13 July 2010

## Data Availability

Not applicable.

## Supplementary Materials

The following supporting information can be downloaded at: https://www.mdpi.com/article/10.3390/microorganisms10081571/s1, Figure S1: A map showing the locations of sampling; Table S1: Set primers and PCR conditions used in the study [94,95,96,97,98,99]; Table S2: Morphological and morphometric data of TAU-MAC strains; Table S3: Similarity of selected Chlorophyta strains from Sphaeropleales based on 18S rRNA gene sequences. TAU-MAC isolates are indicated in bold; Table S4: Similarity of selected Chlorophyta strains from Sphaeropleales based on ITS region sequences. TAU-MAC isolates are indicated in bold; Table S5: Similarity of selected Chlorophyta strains from Sphaeropleales based on *rbcL* gene sequences. TAU-MAC isolates are indicated in bold; Table S6: Similarity of selected Chlorophyta strains from Selenastraceae based on *rbcL* gene sequences. TAU-MAC isolates are indicated in bold; Table S7: Similarity of selected Chlorophyta strains from Chaetophorales based on 18S rRNA gene sequences. TAU-MAC isolates are indicated in bold; Table S8: Similarity of selected Chlorophyta strains from Chaetophorales based on ITS region sequences. TAU-MAC isolates are indicated in bold; Table S9: Similarity of selected Chlorophyta strains from Chlorellales based on 18S rRNA gene sequences. TAU-MAC isolates are indicated in bold; Table S10: Similarity of selected Chlorophyta strains from Chlorellales based on ITS region sequences. TAU-MAC isolates are indicated in bold; Table S11: Similarity of selected Chlorophyta strains from Chlorellales based on *rbcL* gene sequences. TAU-MAC isolates are indicated in bold; Table S12: Similarity of selected Chlorophyta strains from Chlamydomonadales based on 18S rRNA gene sequences. TAU-MAC isolates are indicated in bold; Table S13: Similarity of selected Chlorophyta strains from Chlamydomonadales based on ITS region sequences. TAU-MAC isolates are indicated in bold; Table S14: Similarity of selected Chlorophyta strains from Chlamydomonadales based on *rbcL* gene sequences. TAU-MAC isolates are indicated in bold.
