# Supplementary material for: Uncovering New Diversity of Photosynthetic Microorganisms from the Mediterranean Region
Source: Microorganisms. 2022 Aug 4;10(8):1571. doi: 10.3390/microorganisms10081571 (PMC9416340; doi:10.3390/microorganisms10081571)
Supplement: Supplementary file 1 [file microorganisms-10-01571-s001.zip › microorganisms-1758720-supplementary.pdf]

Supplementary material

Uncovering New Diversity of Photosynthetic Microorganisms from the Mediterranean Region

Urania Lortou [1], Emmanuel Panteris [1] and Spyros Gkelis [1,*]

[1] Faculty of Sciences, School of Biology, Department of Botany, Aristotle University of Thessaloniki, GR-541 24 Thessaloniki, Greece
* Correspondence: sgkelis@bio.auth.gr; Tel.: +302310-998083

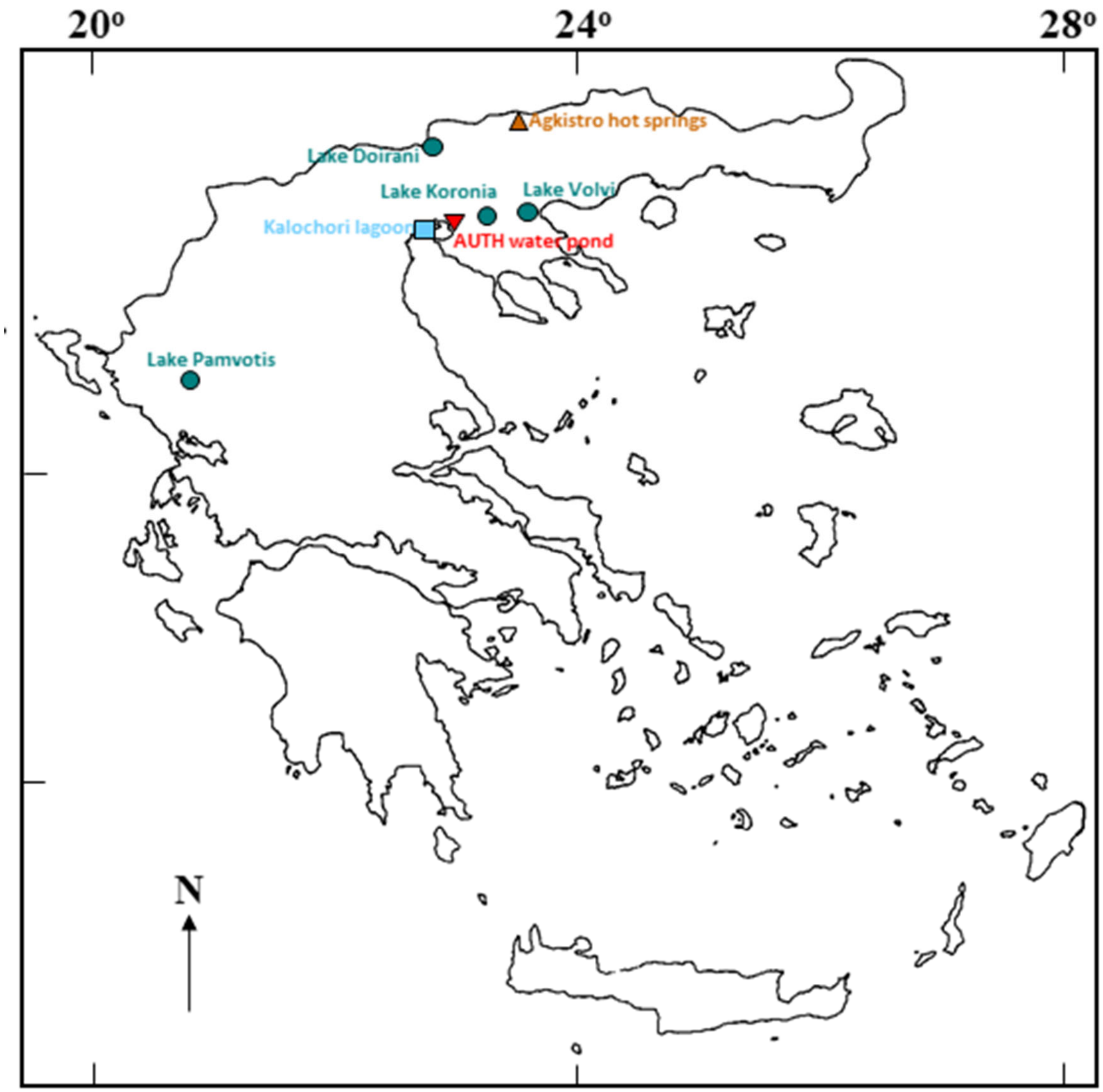

Figure S1: Map of Greece showing the locations of sampling.

Table S1: Set primers and PCR conditions used in the study.

Primer	Target gene-region	Sequence (5′–3′)	Reference	PCR Conditions
NS1F	18S	GTAGTCATATGCTTGTCTC	[94]	initial denaturation step at 95°C for 5 min, followed by 35 cycles consisting of denaturation at 94°C for 30 sec, annealing at 51-54°C for 30 sec and elongation at 72°C for 120 sec; a final 10-min elongation step at 72°C was included.
18L-R		CACCTACGGAAACCTTGTTACGAC TT		
18S-F		AACCTGGTTGATCCTGCCAGT	[95]	initial denaturation step at 94°C for 5 min, followed by 32 cycles consisting of denaturation at 94°C for 50 sec, annealing at 53-56°C for 60 sec and elongation at 72°C for 90 sec; a final 10-min elongation step at 72°C was included.
18S-R		TGATCCTTCTGCAGGTTCACCTACG		
chloroF		TGGCCTATCTTGTTGGTCTGT	[96]	initial denaturation step at 94°C for 3 min, followed by 35 cycles consisting of denaturation at 94°C for 1 min annealing at 57-60°C for 1 min and elongation at 72°C for 1 min; a final 10-min elongation step at 72°C was included.
chloroR		GAATCAACCTGACAAGGCAAC		
NS7m	ITS	GGCAATAACAGGTCTGT	[94]	initial denaturation step at 94°C for 5 min, followed by 32 cycles consisting of denaturation at 94°C for 50 sec, annealing at 42-45°C for 1 min and elongation at 72°C for 90 sec; a final 10-min elongation step at 72°C was included.
LR1850		CCTCACGGTACTTGTTC		
1800F		ACCTGCGGAAGGATCATTG		initial denaturation step at 94°C for 5 min, followed by 32 cycles consisting of

ITS 4R		TCCTCCGCTTATTGATATGC		denaturation at 94°C for 50 sec, annealing at 42-45°C for 1 min and elongation at 72°C for 90 sec; a final 10-min elongation step at 72°C was included.
ChloroRbcL F		GCNGGTGTWAAAGAYTAYGG	[97]	initial denaturation step at 95°C for 3 min, followed by 32 cycles consisting of denaturation at 95°C for 30 sec, annealing at 50-53°C for 1 min and elongation at 72°C for 90 sec; a final 7-min elongation step at 72°C was included.
ChloroRbcL R		TACCACCWGAAGCWACHGGCA		
M28F		GGTGTTGGATTWAAAGCTGGTGT	[98]	initial denaturation step at 94°C for 2 min, followed by 30 cycles consisting of denaturation at 94°C for 75 sec, annealing at 54-57°C for 2 min and elongation at 72°C for 120 sec; a final 7-min elongation step at 72°C was included.
M1390R	*rbcL*	CTTTCCAAAYTTCACAAGCAGCAG		
rbcL1F		GCTGGTGTTAAAGATTATCG	[99]	initial denaturation step at 95°C for 10 min, 35 cycles consisting of denaturation at 94°C for 1 min, annealing at 47-53°C for 45 sec and elongation at 72°C for 150 sec; a final 7-min elongation step at 72°C was included.
rbcL23F		ATACGTGAATACCACCAGAAGC		
rbcL8F		GGTCTTTCAGCTAAAAACTACGG		
rbcL4R		GAAAATGAAACGGTCTCTCC		

Table S2: Morphological and morphometric data of TAU-MAC strains

Strain TAU-MAC	Description	Figure	Taxonomic Assignment
0910, 1010	Colonial green algae having flat colonies that consist of two or four cells linearly arranged along their long axes. Coenobia four-celled most frequent, but also two-celled and eight-celled were observed. Cell dimensions 8–14 μm long and 3–7 μm wide, one chloroplast with one big pyrenoid covered by starch plates, short subpolar spines and small lateral. Starch and lipid accumulate in age cultures. Under light microscopy ribs and rosettes were observed. Asexual reproduction by aplanosporogenesis.	5, 7	*Desmodesmus* sp.
1210	Solitary cells without mucilage, lunate to sigmoid, narrow or acute at the ends, arched or curved. Rarely, colony formation observed by one end attached in mucilage. Cells dimension 5-28μm x 1-7μm. One parietal chloroplast with an embedded pyrenoid naked, without starch cover. Asexual reproduction by autospores.	6, 7	*Monoraphidium* sp.
3310	Ellipsoidal to spherical solitary young cells 4–9 μm in diameter. Young cells contain one nucleus, one parietal chloroplast with one big pyrenoid covered with starch envelope. Mature cells ovoid to irregular in shape, >20 μm in dimension, they organized into dyads, tetrads, or packets resulting from desmoschisis. Chloroplast of mature cells is spongy possessing one to five pyrenoids surrounded by a sheath of starch plates. Asexual reproduction is possible by desmoschisis or zoospores and aplanospores.	6, 8	*Spongiosarcinospis limneus*
3510	Solitary vegetative cells spherical to irregular form, 7-16μm diameter with the ability to form cell aggregates. Sometimes arranged in colonies of randomly distributed	6, 8	*Lilaea pamvotia*

	cells. Chloroplast cup-shaped to reticulate with eyespot and one or several pyrenoids surrounded by starch plates. Single nucleus or multiple nuclei directly before reproduction by aplanospores and may zoospores. Starch and plastoglobuli accumulate in chloroplasts and cytoplasmic oil bodies in age cultures. Thylakoids in bundles of different size and thickness. Asexual reproduction by aplanospores and may zoospores.		
0215	Filaments unbranched and indefinite in length. Cells uninucleate, cylindrical and elongated, closely adherent to one another. Cell diameter increases with the age of a filament from 4 to 10 μm and 7-32 μm long. Chloroplast parietal and band-like, containing more than one pyrenoid. Most of the filaments possess a pointed apical cell at the free end which exhibit a different degree of tapering (acuminate, apiculate or attenuate) and a holdfast for attachment. The morphology of the holdfast ranges from small and colorless to massive and dark brownish-red. Asexual reproduction takes place by aplanosporogenesis or zoosporogenesis.	6, 7	*Uronema trentonense*
0415	Cells ovoid and elongated, 5–13 μm long and 3–7 μm wide. Chloroplast with one large pyrenoid. The four-celled coenobia were predominant, followed by two-celled and solitary cells. Lateral spines longer in outer and shorter in inner cells. Asexual reproduction by aplanosporogenesis.	5	*Desmodesmus subspicatus*
0515	Solitary vegetative cells spherical to irregular form, 9-18 μm diameter, covered with mucilaginous sheath, with the ability to form cell aggregates. Sometimes arranged in colonies of randomly distributed cells. Chloroplast parietal to reticulate with eyespot and one big pyrenoid surrounded by starch plates. Single nucleus. Starch and lipid droplets (plastoglobuli) accumulate in chloroplasts	6, 8	*Akraea chliaropsychia*

	in age cultures. Thylakoids in bundles of different size and thickness. Asexual reproduction by aplanospores and may zoospores.		
1817<br>2017<br>2617	Cells spherical to ovoid, non-motile, 3-9 μm in diameter. Simple structure. Mostly solitary, sometimes arranged in colonies of randomly distributed cells. Chloroplast single, cup-shaped or parietal with an embedded big pyrenoid covered by a starch sheath. In old cultures, accumulation of starch plates and lipid droplets in chloroplast and oil bodies in cytoplasm. Thylakoids arranged in bundles of different size and thickness. Propagation by aplanosporogenesis or cell division of mother cell into two daughter cells. No spines, flagella or cell wall ornaments were observed.	5, 7	*Desmodesmus* sp.
1917<br>2517	Cells ovoid and elongated, with one or two clearly visible pyrenoids surrounded by a sheath of starch plates. Coenobia two (most frequent), four or eight celled in one row, surrounded by mucilage. Cells had dimensions approximately 5-11 x 3-5 μm. Cells with spines were not observed. Asexual reproduction by division of mother cell into two daughter cells.	5	*Desmodesmus multivariabilis*
2117	Cells ovoid to ellipsoidal, 3-9 x 2-6 μm. Chloroplast with one clearly visible pyrenoid surrounded by a sheath of starch plates. The single, spineless cells were dominant. The rare four-celled coenobia were always spined usually with three polar and one lateral spine in the outer cells. Reproduction by autospores.	5	*Desmodesmus abundans*
2217	Cells ovoid to spheroidal, microscopic, approximate 2-8 μm in diameter. Chloroplast single, cup-shaped or parietal, pyrenoid absent. Young cells ellipsoidal, becoming spherical at maturity. Reproduction by aplanospores, two-four autospores per sporangium.	6, 7	*Ava limnothalassea*

2717	Cells spherical to ovoid, 3-9 μm in diameter. Rarely, colonies of randomly distributed cells were observed. Chloroplast parietal with one big pyrenoid surrounded by a sheath of starch plates. Asexual reproduction by aplanosporogenesis.	5	*Desmodesmus abundans*
3617	Cells spherical or oval, microscopic, approximate 2-9 μm in diameter. One lateral cup-shaped or discoid chloroplast, lacking a pyrenoid. Starch grains sometimes observed. Cell wall thin with bristle-like hair in the surface. Propagation by autosporulation.	6, 10	*Nomia picochloropsia*
3917	Cells non-motile, irregularly spherical to broadly ovoid arranged in coenobia of randomly distributed cells, surrounded by mucilage envelope. Most frequent observed solitary cells. Cells dimensions 5 μm -up to 30 μm and some very big cells up to 40μm also observed. Chloroplast parietal and net-like in adult cells with one big pyrenoid surrounded by starch. Age cultures accumulate oil bodies and pigments that turn the color of cells from green to orange/ red. Asexual reproduction by autospores organized into coenobia.	6	*Asterarcys quadricellulare*

Table S3: Similarity of selected Chlorophyta strains from Sphaeropleales based on 18S rRNA gene sequences. TAU-MAC isolates are indicated in bold.

	1	2	3	4	5	6	7	8	9	10	11	12	13	14	15	16	17	18	19	20	21	22	23	24	25
1. AY846382.1	100																								
**2. TAU-MAC 1210**	99.3	100																							
3. AB917098.1	98.8	99.4	100																						
4. Y16937.1	98.9	99.3	99.8	100																					
5. KT833577.1	98.8	99.3	99.8	99.8	100																				
6. JQ315573.1	93.5	93.2	93.3	93.3	93.3	100																			
7. AY197638.1	93.8	93.3	93.6	93.6	93.6	98.3	100																		
**8. TAU-MAC 2617**	93.6	93.2	93.4	93.4	93.4	98.1	99.5	100																	
**9. TAU-MAC 1817**	93.8	93.3	93.6	93.5	93.6	98.1	99.4	99.6	100																
**10. TAU-MAC 2017**	93.7	93.3	93.5	93.5	93.5	98.1	99.5	99.9	99.7	100															
11. AY197627.1	94.1	93.6	93.8	93.9	93.9	98.4	99.3	99.0	98.9	98.9	100														
12. AY197634.1	94.2	93.8	93.9	93.8	93.9	98.4	99.0	98.7	98.8	98.7	99.3	100													
**13. TAU-MAC 2717**	93.0	92.9	92.8	92.7	92.8	98.3	98.0	97.9	97.9	98.0	98.1	97.9	100												
**14. TAU-MAC 0910**	93.4	92.8	93.0	93.1	93.1	98.8	98.2	98.0	98.1	98.0	98.4	98.3	98.2	100											
**15. TAU-MAC 1010**	93.4	92.8	93.0	93.1	93.1	98.8	98.2	98.0	98.1	98.0	98.4	98.3	98.2	100	100										
16. AB917139.1	93.5	93.1	87.2	93.1	93.2	94.6	98.8	98.3	98.4	98.3	98.9	98.7	98.8	94.4	94.4	100									
17. KU175228.1	93.6	93.1	87.2	93.3	93.4	99.2	98.6	98.3	98.4	98.4	98.6	98.6	98.5	98.9	98.9	92.8	100								
18. TAU-MAC 1917	93.4	93.0	93.2	93.1	93.2	99.4	98.0	97.8	97.9	97.9	98.2	98.0	98.5	98.5	98.5	98.7	98.8	100							
19. TAU-MAC 2517	93.4	93.0	93.2	93.1	93.2	99.4	98.0	97.8	97.9	97.9	98.2	98.0	98.5	98.5	98.5	98.7	98.8	100	100						
20. MH683854.1	92.6	92.3	92.3	92.3	92.3	99.1	97.5	97.6	97.3	97.3	97.7	97.5	98.3	98.2	98.2	98.2	98.6	99.8	99.8	100					
21. MG022724.1	93.8	93.5	93.6	93.5	93.6	99.0	98.5	98.3	98.4	98.4	98.6	98.5	99.5	98.8	98.8	99.2	99.2	98.7	98.7	98.7	100				
22. AB917128.1	93.8	93.5	93.6	93.5	93.6	99.0	98.5	98.3	98.4	98.4	98.6	98.5	99.5	98.8	98.8	99.2	99.2	98.7	98.7	98.7	98.7	100			
23. MW471025.1	93.8	93.5	93.6	93.5	93.6	99.0	98.5	98.3	98.4	98.4	98.6	98.5	99.5	98.8	98.8	99.2	99.2	98.7	98.7	98.7	100	100	100		
24. EU910612.1	93.8	93.3	93.6	93.4	93.6	99.3	98.6	98.5	98.5	98.4	98.5	98.5	98.8	98.9	98.9	99.3	99.2	99.0	99.0	98.5	99.3	99.3	99.3	100	
25. MK764918.1	93.5	93.0	93.2	93.1	93.2	99.1	98.5	98.3	98.3	98.3	98.8	98.4	98.6	98.9	98.9	99.4	99.1	99.0	99.0	98.6	99.3	99.3	99.3	99.4	100

Table S4: Similarity of selected Chlorophyta strains from Sphaeropleales based on ITS region sequences. TAU-MAC isolates are indicated in bold.

	1	2	3	4	5	6	7	8	9	10	11	12	13	14	15	16	17	18	19	20	21	22	23	24	25
1. MH203022.1	100																								
**2. TAU-MAC 1210**	91.9	100																							
3. MH010848.1	90.7	94.6	100																						
4. KT274017.1	90.1	94.9	97.3	100																					
5. JX456463.1	90.4	94.4	97.2	97.3	100																				
**6. TAU-MAC 2717**	76.1	75.7	75.7	75.7	75.0	100																			
7. AB917128.1	76.1	75.7	75.5	75.5	75.2	98.4	100																		
8. MW471025.1	76.1	75.7	75.5	75.5	75.2	98.4	100	100																	
9. KT778088.1p	76.1	75.6	75.3	75.4	75.0	98.2	99.8	99.8	100																
10. MF357034.1	76.7	76.3	75.4	75.3	74.7	91.9	91.4	91.4	91.2	100															
11. DQ417531.1	77.2	76.6	75.4	75.8	75.3	91.5	91.4	91.4	91.2	98.9	100														
12. MN738557.1	77.0	76.4	75.7	75.7	75.0	91.5	91.3	91.3	91.1	98.9	100	100													
**13. TAU-MAC 0910**	77.2	76.6	75.9	75.8	75.3	91.6	91.4	91.4	91.3	98.5	99.7	99.7	100												
**14. TAU-MAC 1010**	77.4	76.8	76.1	76	75.5	91.8	91.6	91.6	91.4	98.6	99.8	99.8	99.9	100											
15. DQ417525.1	76.6	74.9	75.3	74.7	74.2	91.0	90.2	90.2	90.2	93.5	94	93.9	94.0	94.1	100										
16. MT603589.1	76.2	74.6	75.0	74.5	74.0	90.9	89.9	89.9	89.9	93.5	93.8	93.8	93.5	93.6	98.9	100									
**17. TAU-MAC 2517**	76.2	74.6	75.0	74.5	74.0	91.1	90.1	90.1	90.1	93.3	93.8	93.8	93.6	93.8	98.9	99.8	100								
18. MH010854.1	76.2	74.6	75.0	74.5	74.0	91.1	90.1	90.1	90.1	93.3	93.8	93.8	93.6	93.8	98.9	99.8	100	100							
**19. TAU-MAC 1917**	76.0	74.5	74.9	74.3	73.8	90.9	89.9	89.9	89.9	93.2	93.8	93.6	93.4	93.5	98.9	99.8	99.8	99.8	100						
20. DQ417556.1	76.4	75.4	75.5	76.3	74.9	79.3	79.1	79.3	79.3	80.6	80.6	80.6	80.4	80.4	80.0	80.5	80.5	80.5	80.5	100					
21. DQ417533.1	77.9	76.7	76.9	77.3	76.9	81.5	81.3	81.3	81.3	82.0	82.1	81.7	81.8	81.8	82.1	82.1	82.1	82.1	82.3	96.7	100				
**22. TAU-MAC 1817**	75.2	73.5	72.9	74.1	72.8	78.5	78.3	78.3	78.3	79.2	78.5	78.5	78.8	78.8	77.6	77.8	77.6	77.4	82.8	84.0	100	100			
**23. TAU-MAC 2017**	75.2	73.5	72.9	74.1	72.8	78.5	78.3	78.3	78.3	79.2	78.5	78.5	78.8	78.8	77.6	77.8	77.6	77.4	82.8	84.0	100	100	100		
**24. TAU-MAC 2617**	75.2	73.5	72.9	74.1	72.8	78.5	78.3	78.3	78.3	79.2	78.5	78.5	78.8	78.8	77.6	77.8	77.6	77.6	77.4	82.8	84.0	100	100	100	
25. KT445862.2	74.9	73.8	72.9	73.7	72.1	79.0	78.7	78.7	78.7	79.7	78.9	78.8	79.0	79.0	78.5	78.5	78.5	78.5	84.1	84.4	87.9	87.9	87.9	100	100

Table S5: Similarity of selected Chlorophyta strains from Sphaeropleales based on *rbcL* gene sequences. TAU-MAC isolates are indicated in bold.

	1	2	3	4	5	6	7	8	9	10	11	12	13	14	15	16	17
1. MK257133.1	100																
2. MF278351.1	91.5	100															
**3. TAU-MAC 2717**	91.3	94.8	100														
4. KF975604.1	90.8	91.8	92.7	100													
5. KT777973.1	91.2	92.2	93.7	97.4	100												
6. KT777963.1	91.3	92.3	93.8	97.5	99.9	100											
**7. TAU-MAC 1817**	90.8	91.7	93.2	96.7	98.9	99	100										
**8. TAU-MAC 2617**	91.0	92.0	93.7	97.2	99.4	99.5	99.5	100									
**9. TAU-MAC 2017**	90.7	91.8	93.4	97	99.1	99.8	99.3	99.7	100								
**10. TAU-MAC 2517**	91.0	93.9	95.1	94.1	94.5	94.6	93.8	94.2	93.3	100							
**11. TAU-MAC 1917**	91.1	94	95.1	94.2	94.6	94.7	93.9	94.3	94.0	99.9	100						
12. EF012704.1	91.6	93.9	94.8	94.1	94.9	95	94.1	94.6	94.3	97.7	97.8	100					
13. KF975595.1	92.0	94	95	94.8	95.3	95.5	94.6	95.1	94.8	97.2	97.3	97.7	100				
14. GU192431.1	91.2	93.5	94.4	93.8	94.7	94.8	94	94.4	94.1	97.2	97.3	98.4	98.4	100			
15. MK257140.1	93.2	93.2	94	93.3	93.5	93.6	92.9	93.2	92.9	94.2	95.1	95.1	95.4	94.8	100		
**16. TAU-MAC 1010**	93.2	92.8	93.6	92.4	93.1	93.2	92.7	92.9	92.7	94.5	94.6	94.7	95.1	94.4	96.5	100	
**17. TAU-MAC 0910**	92.9	92.3	93.3	92.3	92.9	92.9	92.4	92.7	92.4	94.2	94.4	94.4	94.8	94.1	96.1	99.5	100

Table S6: Similarity of selected Chlorophyta strains from Selenastraceae based on *rbcL* gene sequences. TAU-MAC isolates are indicated in bold.

	1	2	3	4	5	6	7	8	9
1. KT355767.1	100								
2. KT355752.1	93.5	100							
3. KT355750.1	91.5	93.0	100						
4. **TAU-MAC 1210**	93.0	95.0	95.5	100					
5. KT355751.1	93.7	94.2	95.2	96.1	100				
6. KT833568.1	94.2	94.1	95.2	96.4	96.9	100			
7. KT833562.1	93.8	94.7	95.3	96.1	96.1	97.3	100		
8. KT833566.1	92.9	93.9	92.9	93.0	93.4	92.7	92.5	100	
9. LC472548.1	92.7	93.6	93.0	93.8	93.6	93.2	93.3	94.0	100

Table S7: Similarity of selected Chlorophyta strains from Chaetophorales based on 18S rRNA gene sequences. TAU-MAC isolates are indicated in bold.

	1	2	3	4	5	6	7	8	9	10
**1. TAU-MAC 0215**	100									
2. KM020161.1	100	100								
3. KM020181.1	100	100	100							
4. FN824390.1	100	99.9	99.9	100						
5. AF182821.1	99.9	99.9	99.9	99.9	100					
6. KM020091.1	99.8	99.8	99.8	99.8	99.8	100				
7. FN824393.1	99.5	99.5	99.5	99.6	99.5	99.4	100			
8. MK250084.1	99.5	99.5	99.5	99.5	99.5	99.5	100	100		
9. FN824391.1	99.5	99.5	99.5	99.5	99.5	99.5	99.3	99.3	100	
10. MH683840.1	99.5	99.5	99.5	99.5	99.4	99.4	99.5	99.5	99.2	100

Table S8: Similarity of selected Chlorophyta strains from Chaetophorales based on ITS region sequences. TAU-MAC isolates are indicated in bold.

	1	2	3	4	5
**1. TAU-MAC 0215**	100				
2. JX092263.1	98	100			
3. KT308082.2	97.8	99.5	100		
4. MH683943.1	87.3	87.8	87.9	100	
5. FR865743.1	89.9	90.9	90.6	86.1	100

Table S9: Similarity of selected Chlorophyta strains from Chlorellales based on 18S rRNA gene sequences. TAU-MAC isolates are indicated in bold.

	1	2	3	4	5	6	7	8	9	10
**1. TAU-MAC 2217**	100									
**2. TAU-MAC 3617**	96.6	100								
3. JN086476.1	96.5	99.8	100							
4. KF791551.1	96.5	99.8	99.8	100						
5. MH683906.1	96.6	99.9	99.9	99.9	100					
6. KM020037.1	96.2	97.3	97.2	97.2	97.3	100				
7. AY195968.1	96.8	97.7	97.5	97.5	97.6	97.7	100			
8. AJ439399.2	96.6	97.4	97.2	97.2	97.3	97.3	98.7	100		
9. LN610705.1	96.7	97.4	97.3	97.3	97.4	97.4	98.7	99.9	100	
10. KM020039.1	96.6	97.3	97.2	97.2	97.3	97.3	98.6	99.9	99.9	100

Table S10: Similarity of selected Chlorophyta strains from Chlorellales based on ITS region sequences. TAU-MAC isolates are indicated in bold.

	1	2	3	4	5	6	7	8
1. KM020039.1	100							
2. LN610705.1	99.6	100						
**3. TAU-MAC 2217**	80.7	81.0	100					
4. KJ676113.1	74.4	74.8	83.2	100				
5. AB731601.1	74.4	74.8	83.2	100	100			
6. MK764925.1	74.6	74.9	83.4	100	100	100		
7. MK643407.1	74.6	74.9	83.4	100	100	100	100	
8. KP726221.1	74.6	74.9	83.4	100	100	100	100	100

Table S11: Similarity of selected Chlorophyta strains from Chlorellales based on *rbcL* gene sequences. TAU-MAC isolates are indicated in bold.

	1	2	3	4	5	6	7	8	9
**1. TAU-MAC 3617**	100								
2. KP202854.1	99.5	100							
3. MG552671.1	92.4	92.4	100						
**4. TAU-MAC 2217**	89.8	89.5	90.0	100					
5. AB240145.1	90.8	90.6	89	90.7	100				
6. MN128434.1	90.9	90.6	89.4	90.5	99.1	100			
7. MK257138.1	90.4	90.1	91.1	91.8	91.8	91.9	100		
8. KM462888.1	90.3	90.0	91.3	90.7	90.9	90.9	93.1	100	
9. AB383150.1	90.8	90.6	90.6	90.5	91.1	91.1	92.8	94.1	100

Table S12: Similarity of selected Chlorophyta strains from Chlamydomonadales based on 18S rRNA gene sequences. TAU-MAC isolates are indicated in bold.

	1	2	3	4	5	6	7	8	9	10	11
1. EF159950.1	100										
2. KF144170.1	99.1	100									
**3. TAU-MAC 0515**	99.2	99.9	100								
4. MH651272.1	99.2	99.9	100	100							
5. KF791539.1	96.5	96.4	96.4	96.4	100						
6. MF687231.1	96.4	96.4	96.4	96.4	97.9	100					
7. AY220603.1	96.2	96.0	96.1	96.1	97.6	99.6	100				
**8. TAU-MAC 3310**	96.8	97.2	97.3	97.3	98.1	99.8	99.9	100			
9. AY220598.1	96.2	96.2	96.2	96.2	97.7	99.8	99.8	99.9	100		
**10. TAU-MAC 3510**	93.7	94.1	94.1	94.1	95.0	95.3	95.2	95.3	95.3	100	
11. KF791546.1	92.6	92.8	92.7	92.7	93.7	93.9	93.8	94.6	94	96.6	100

Table S13: Similarity of selected Chlorophyta strains from Chlamydomonadales based on ITS region sequences. TAU-MAC isolates are indicated in bold.

	1	2	3	4	5	6	7	8	9	10	11	12	13
**1. TAU-MAC 3510**	100												
2. KT308081.2	82.5	100											
3. KP406624.1	72.1	71	100										
**4. TAU-MAC 3310**	71	71.7	87.6	100									
5. MH068690.1	66.3	68.7	87.1	93.2	100								
6. MH684014.1	71.0	72.9	69.1	73.2	69.3	100							
7. MH651305.1	70.3	72.1	68.4	73.9	69.3	99.7	100						
8. MH619608.1	72.5	73.2	72.7	73.5	70.1	77.2	76.6	100					
9. KT308086.2	74.8	74.6	76.7	76.6	75.5	78.7	78.1	80.2	100				
10. MH651319.1	75.6	74.2	72.7	73.3	71.8	78.8	78.2	80.4	85.5	100			
**11. TAU-MAC 0515**	76.2	74.4	73.9	75.1	73.4	78.7	78.4	80.5	86.3	99.8	100		
12. JQ315797.1	77.5	75	74.2	75.1	73.6	78.8	78.3	80.4	86.5	99.7	99.8	100	
13. JQ315798.1	77.4	76.4	74.9	76.6	74.3	79.9	79.5	81.5	84.9	99.6	99.8	100	100

Table S14: Similarity of selected Chlorophyta strains from Chlamydomonadales based on *rbcL* gene sequences. TAU-MAC isolates are indicated in bold.

	1	2	3	4	5	6	7	8	9	10	11
1. KJ635640.1	100										
**2. TAU-MAC 3310**	92.7	100									
3. MK257143.1	92.6	96.3	100								
4. HE860265.1	91.7	92	93	100							
5. HE860264.1	91.8	91.9	92.6	98.4	100						
**6. TAU-MAC 3510**	92.5	91.7	92.2	93.1	93.8	100					
7. JN880462.1	91.8	91.5	92.0	92.7	93.0	94.1	100				
8. JN880463.1	91.7	92.0	92.2	93.5	93.7	94.2	98.2	100			
9. JN880465.1	91.7	91.5	92.2	92.8	93.2	93.9	98.4	98.9	100		
**10. TAU-MAC 0515**	90.7	90.2	90.6	90.3	90.7	90.7	92.2	92.3	92.6	100	
11. EF113426.1	91.5	91.1	91.4	91.5	92.1	92.2	92.4	92.6	93.1	93.2	100
